# Compact CRISPR genetic screens enabled by improved guide RNA library cloning

**DOI:** 10.1186/s13059-023-03132-3

**Published:** 2024-01-19

**Authors:** Seok-Jin Heo, Lauren D. Enriquez, Scot Federman, Amy Y. Chang, Rachel Mace, Kaivalya Shevade, Phuong Nguyen, Adam J. Litterman, Shawn Shafer, Laralynne Przybyla, Eric D. Chow

**Affiliations:** 1 Laboratory for Genomics Research, San Francisco, CA 94158 USA; 2 Department of Biochemistry and Biophysics, University of California, San Francisco, San Francisco, CA 94158 USA; 3 GSK, San Francisco, CA 94158 USA

**Keywords:** CRISPR screening, Library cloning, Guide library, iPSC-derived CRISPR screening, Primary cell CRISPR screening

## Abstract

CRISPR genome editing approaches theoretically enable researchers to define the function of each human gene in specific cell types, but challenges remain to efficiently perform genetic perturbations in relevant models. In this work, we develop a library cloning protocol that increases sgRNA uniformity and greatly reduces bias in existing genome-wide libraries. We demonstrate that our libraries can achieve equivalent or better statistical power compared to previously reported screens using an order of magnitude fewer cells. This improved cloning protocol enables genome-scale CRISPR screens in technically challenging cell models and screen formats.

**Supplementary Information:**

The online version contains supplementary material available at 10.1186/s13059-023-03132-3.

## Background

The human genome project produced the first assembled human genome over 20 years ago [1, 2]. Genomic sequencing efforts reveal genes and genetic variation associated with disease but for the most part do not reveal gene function. As such, functional genomics efforts have been critical to assign function to the roughly 20,000 human protein-coding genes identified. In the past decade, CRISPR (clustered regularly interspaced short palindromic repeats)-based screens have increased the ease of genome-wide genetic screens, allowing researchers to find new components of biological pathways, assign mechanism to existing drugs, identify novel therapeutic targets, and uncover synergistic genetic relationships [3–7]. However, due to the size of genome-wide guide libraries (20,000–200,000 + elements) and typical cell coverage required (500–1000-fold) to accurately quantify gene hits and average out phenotype-independent variability across the population, each screen requires tens to hundreds of millions of cells per sample [8–12]. This requirement poses a logistical challenge for cell models where large-scale culturing is difficult, such as adherent cell lines or growth-limited models such as primary and differentiated cell lines [13–15].

A major factor that influences cell coverage is library uniformity, as larger variation in individual guide RNA abundance requires higher cell coverage to reliably measure low-abundance guides. In this work, we report optimizations to several steps in CRISPR guide library cloning that significantly decrease guide representation bias, allowing for screening at lower cell coverage [16]. Improvements were made in the following areas. First, ordering guide oligos in both forward and reverse complement orientations to counteract sequence-specific biases in oligo synthesis [17]. Second, decreasing the number of PCR cycles used to prepare inserts to avoid over amplification to maintain library uniformity. Lastly, working at low temperatures during insert preparation to reduce biased dropout of inserts with lower melting temperatures (*T*_m_). We used these optimizations to clone new versions of published genome-wide CRISPRi and CRISPRa libraries [18] and achieved more uniform guide distributions, as evidenced by reduced skew ratios compared to the libraries available on Addgene (#83969 and #83978; referred to as legacy throughout the text).

With the improved CRISPRi library, we demonstrate comparable performance in survival screens at 100 versus 1000-fold coverage. In a survival screen coupled with treatment with the tyrosine kinase inhibitor dasatinib in K562 cells, we observe more hits in expected pathways compared to those identified in parallel screens run using a publicly available CRISPRi library as well as a previously reported CRISPRn screen. Lastly, through a transduction titration experiment, we demonstrate the feasibility of performing screens at 50-fold cell coverage, facilitating genome-wide screens requiring only 5 million cells per sample for a 100,000-guide library. This level of coverage will enable researchers to use more sophisticated and biologically relevant readouts such as FACS-based, imaging, and single-cell sequencing approaches and model systems such as adherent cells, iPSC-derived cells, and primary cells, which were previously challenging or impossible to work with at genome-scale. The cloning methods described here are generalizable across any library cloned from oligo pools, including guide libraries for any type of CRISPR based approach including nuclease, base editing [19], prime editing [20], CRISPRoff [21], and RNA editing applications, and are applicable across a range of Cas enzymes including Cas9, Cas12, and Cas13.

## Results

### Cloning optimization reduces library bias

**Fig. 1:**
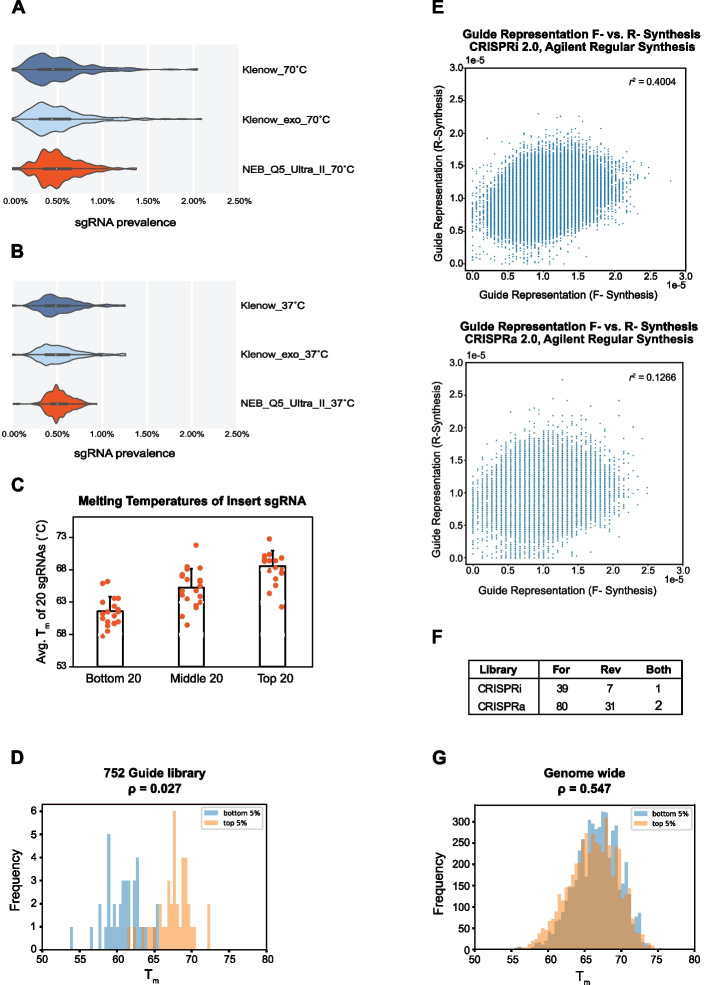
Factors affecting guide cloning uniformity. **A** Violin plots depicting guide abundance distributions of libraries prepared with three different polymerases (Klenow, Klenow exo-, and NEB Q5 Ultra II) and inserts extracted at 70˚C. **B** Violin plots of libraries prepared the three different polymerases and a 37˚C extraction. **C** Melting temperature (*T*_m_) and abundance of the lowest, mid, and top 20 guides in the 752-element pilot library. **D** Mann–Whitney *U* test comparing *T*_m_s of high (top 5%) and low (bottom 5%) abundance guides in the 752-element pilot library. **E** Correlation between forward and reverse complement oligo pools for CRISPRi V2 (top) and CRISPRa V2 (bottom) using a linear least-squares regression. **F** Number of guides missing from the oligos pools in forward, reverse, or combined sets in the CRISPRi V2 and CRISPRa V2 guide libraries. **G** Mann–Whitney *U*-test comparing the *T*_m_s of high (top 5%) and low (bottom 5%) abundance guides in the CRISPRi V2 genome-wide library

To improve library uniformity, we performed a series of cloning optimizations to improve the representation of CRISPR sgRNA libraries using sequences from previously described genome-wide CRISPRi and CRISPRa libraries [18]. To construct these libraries, single-stranded oligo templates are amplified and converted to double stranded inserts that are cloned into a vector. In the CRISPRi/a library cloning protocol, the insert is digested into a 33-bp double-stranded product, gel purified, and ligated into a lentiviral expression vector (pLGR1002). We first examined whether the polymerase used to synthesize double-stranded DNA encoding the sgRNA insert could impact library representation. In a pilot experiment comparing three different polymerases (Klenow, Klenow exo-, and NEB Q5 Ultra II), we observed varying guide representations in a library of 192 sgRNAs, (Fig. 1A) with the most uniform representation observed in inserts prepared with Q5 Ultra II polymerase. To check if the clones contained the expected insert, we performed colony PCR using PCR primers outside of the BstXI and BlpI restriction sites (Additional file 1: Fig. S1A). We observed that many of the individual clones in libraries prepared with all three polymerases had the expected 290-bp band but 40% exhibited additional higher molecular weight bands (Additional file 1: Fig. S1B). Sanger sequencing of plasmids isolated from these colonies showed mixed bases in the spacer region of the sgRNA (Additional file 1: Fig. S1C). We hypothesize that these mixed sequences are derived from transformation with plasmids containing non-complementary hybrid inserts. As predicted from this model, retransformation of these plasmids yielded colonies that gave a single band upon colony PCR (Additional file 1: Fig. S1D).

Next, we tested if a lower 37˚C insert elution during gel purification narrows guide distribution and reduced the formation of hybrid clones. Using the same starting material, we found that the lower elution temperatures increased uniformity and Q5 Ultra II polymerase still performed better than Klenow (Fig. 1B). Furthermore, the lower elution temperature reduced the formation of hybrid clones from 40% to 1.2% (Additional file 1: Fig. S2). We also observed that PCR amplification of oligo pools produced a single narrow band of product, whereas primer extension with Klenow yielded non-specific products that were both smaller and larger than the intended insert (Additional file 1: Fig. S3).

We moved on to a larger pilot library of 752 guide RNAs to further optimize the cloning protocol. Since Q5 Ultra II polymerase performed better than the two different mesophilic polymerases, we sought to determine the effect of additional PCR cycles on guide distribution. While we used NEB Q5 in this study, there are other NGS-optimized polymerases that could provide even better performance. We repeated the library cloning described above with either 1 or 15 cycles of insert PCR. A pairwise comparison indicated similar representations of the 752 gRNAs across the two libraries (Additional file 1: Fig. S4). During this experiment, we observed higher molecular weight smears in some PCR products (Additional file 1: Fig. S5A) that are likely overamplification bubble products that contribute to the hybrid clones observed in earlier experiments. We reduced the number of PCR cycles and optimized template concentration to minimize these undesired products (Additional file 1: Fig. S5B). In this new library, even though the gel-purified insert was eluted at 37˚C, we still observed a relationship between guide abundance and the melting temperature (*T*_m_) of the inserts (Fig. 1C). The average *T*_m_ of the 20 most highly represented gRNAs was higher than 68˚C. In contrast, the average melting temperature of the 20 most lowly represented gRNAs was less than 62˚C. A Mann–Whitney *U* test comparing the lowest and highest 5th percentiles of guide representation (*n*_1_ = 38, *n*_2_ = 38) indicated a statistically significant difference between the *T*_m_ distributions of these two populations (Fig. 1D). These results suggest a 37˚C elution temperature can still bias guide abundance due to *T*_m_ differences. 

We next sought to reduce heterogeneity of guide abundance in the template oligo pool. Specific oligo sequences and motifs can affect yields and many groups order oligo templates in both orientations, but we have not found data that supports this. To confirm the utility of ordering oligos in both orientations, we compared the abundance of oligos in each pool using a single stranded DNA library preparation kit. Using a linear least-squares regression model, we observed a weak correlation between the representation of guide sequences derived from different strand synthesis pools (Fig. 1E). This suggests that ordering oligo templates in both orientations will reduce final library bias. Additionally, we observed a non-overlapping subset of guides missing from oligos synthesized in either orientation (Fig. 1F), indicating reduced dropouts is another benefit or ordering oligos in both orientations.

To test the improved cloning strategy, we cloned two genome-wide guide libraries using our improved protocol. Since a *T*_m_-dependent guide bias was still present with a 37˚C elution, we performed insert gel electrophoresis on ice and reduced the elution temperature to 4˚C. Using oligo pools ordered in both orientations, we recloned the V2 CRISPRi and CRISPRa libraries which respectively contain 103,074 and 101,250 elements (5 sgRNA/gene; guide sequences can be found in Additional File 2) [18]. After sequencing the cloned libraries, we repeated the Mann–Whitney *U* test comparing the melting temperature of the lowest 5% represented CRISPRi guides (*n* = 5151) against the highest 5% represented guides (*n* = 5151) and observed a ρ statistic of 0.547 (Fig. 1G), compared to a *ρ* statistic of 0.027 for 752-guide library prepared with a 37˚C elution (Fig. 1D). The *ρ* statistic values indicate that the lowest 5% most represented guides and the highest 5% represented guides shared a similar underlying distribution, which was not the case in the 752-guide library that used insert templates synthesized in a single orientation and a 37˚C insert elution temperature.

**Fig. 2:**
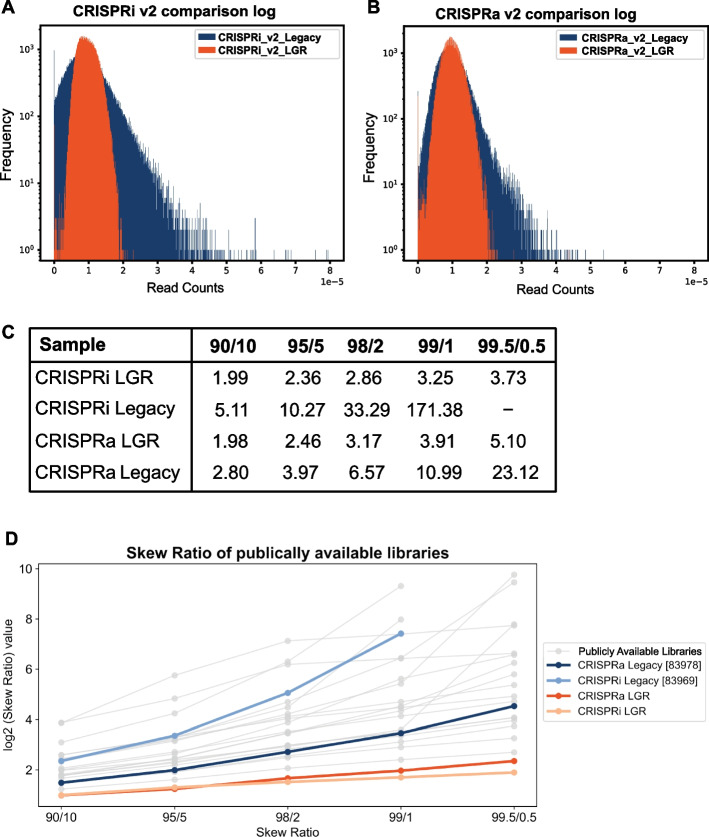
New optimizations in the cloning protocol improve genome-wide guide libraries. **A** Histograms of the CRISPRi V2 legacy library (blue) compared to the optimized CRISPRi V2 LGR library (orange) show that the LGR library has a tighter distribution of sgRNAs. **B** Similarly, the CRISPRa V2 LGR library (orange) shows a tighter distribution than the CRISPRa V2 legacy library (blue). **C** Skew ratio table comparing CRISPRi V2 and CRISPRa V2 LGR libraries to the legacy libraries. **D** Skew ratios of publicly available CRISPR libraries (Addgene catalog numbers available in Additional file 4) at the 90/10, 95/5, 98/2, 99/1, and 99.5/0.5 percentiles compared to the CRISPRi and CRISPRa LGR libraires

When compared to the legacy libraries, both of our libraries show a more uniform distribution (Fig. 2A, B) with fewer dropouts. This result was not due to undersequencing as each sample was sequenced to a depth of 500–2000-fold coverage (Additional file 3). Skew ratios are used to quantitatively compare library uniformity. They are calculated by calculating the ratio of the abundance of guide pairs at different percentiles, with lower ratios indicating more uniform libraries. In a 100,000-element library, a 90/10 skew ratio compares the abundance of the 10,000th top and bottom elements. Our libraries have a 90/10 skew ratio under 2, outperforming the legacy libraries. The uniformity is more evident when comparing skew ratios at the extremes of the distribution (Fig. 2C), where the difference between the top 1% and 99% guides is under 4. Even more impressive, the legacy library was cloned as seven smaller subpools, compared to a single large pool for our library. The skew in the legacy libraries is impacted by a few subpools with larger variance (Tables S1-S4). However, the our newly cloned libraries had lower skew ratios than any individual subpool and demonstrate the benefit of cloning the entire library in a single reaction. Furthermore, when compared to publicly available CRISPR libraries, the LGR libraries were of higher quality across 90/10, 95/5, 98/2, 99/1, and 99.5/0.5 skew ratios (Fig. 2D, Additional file 4). The improvements we have made in our library cloning protocol (Additional file 5) are easy for users to adopt and will consequently result in high quality libraries with more uniform distributions.

### Lower skew library performs well at lower cell coverage

**Fig. 3:**
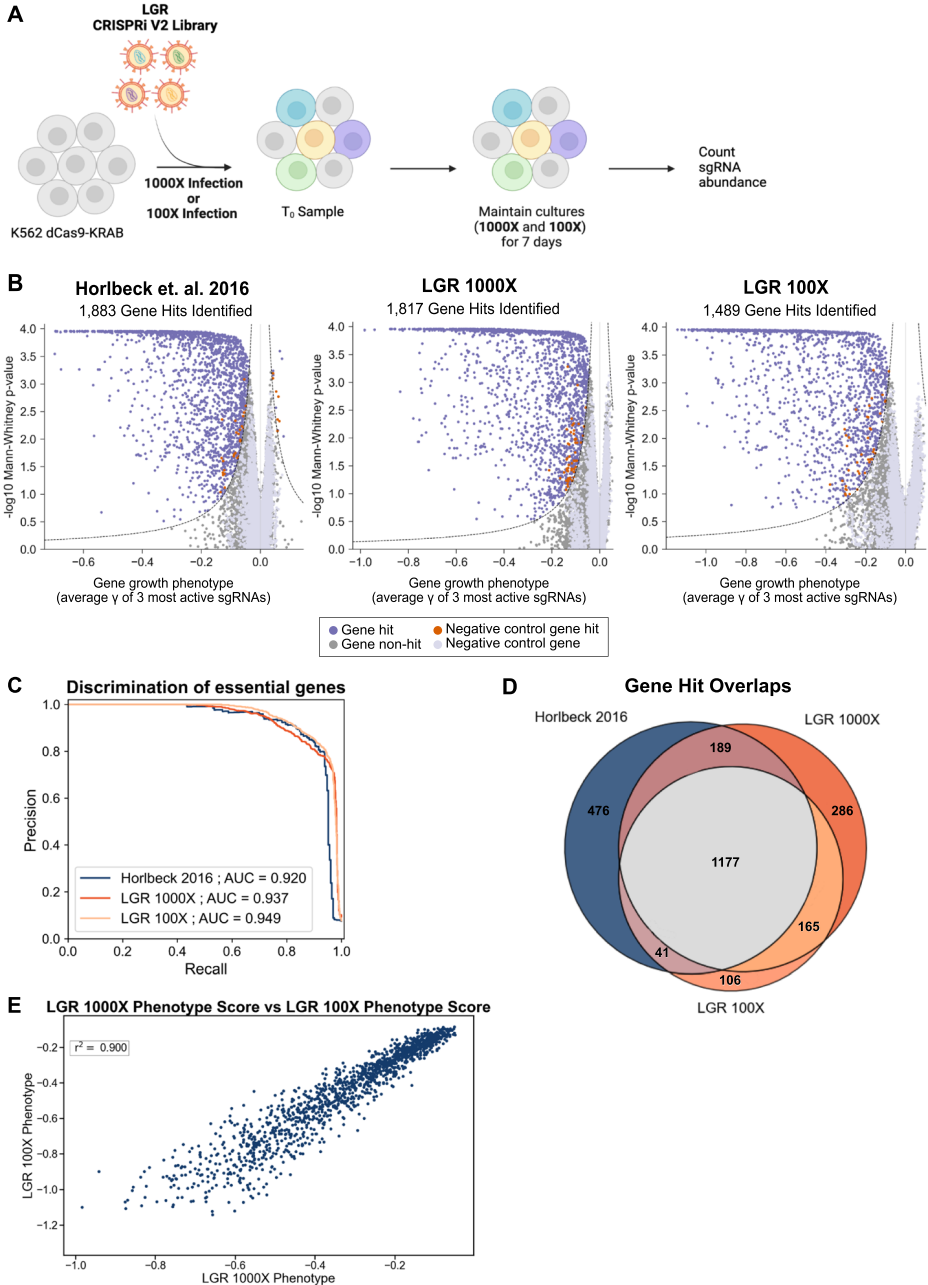
The optimized LGR library performs similarly to the existing legacy library, even at lower cell coverage. **A** Schematic of the CRISPRi V2 survival screen in K562 cells performed with the LGR library at 100- or 1000-fold cell coverage. **B** Comparison of the 1000-fold cell coverage screen performed by Horlbeck et. al (2016) [18] using the CRISPRi V2 legacy library (left) versus the 1000-fold (middle) and 100-fold (right) cell coverage screens performed using the CRISPRi V2 LGR library. **C** Precision-recall analysis of the essential genes identified in each library using BAGEL2 [25, 26] to measure screening quality between the legacy library and the LGR library. The area under the curve (AUC) for each library were as follows: legacy (Horlbeck et. al 2016) [18] 0.920, LGR 1000-fold 0.937, and LGR 100-fold 0.949. **D** A diagram illustrating the amount of overlap in essential genes identified in each screen. **E** A point comparison of the phenotype score of overlapping hits between the CRISPRi V2 LGR 100 and 1000-fold screens

Previous work suggested libraries with 90/10 skew ratios below 2 could be screened at 100-fold cell coverage [22]. To test this, we performed a genome-wide CRISPRi survival screen in the chronic myeloid leukemia cell line K562s expressing dCas9-KRAB transduced and maintained at 100 or 1000-fold guide library coverage (Fig. 3A, Additional file 1: Fig. S6A). Cells were infected at a rate of 20.2–23.7% to minimize cells undergoing multiple transductions. We compared the essential genes identified in our 1000-fold screen to those previously identified [18]. The original study used the expanded library of 10 guides per gene, so we extracted data from the top 5 guides for each gene and analyzed the data with the ScreenProcessing pipeline (refer to “Methods: Computational analysis of screens”) for a direct comparison between the original study and our library. Essential genes are identified by the gamma score, which represents the growth enrichment (log_2_ enrichment) determined by sgRNA read counts between the untreated sample and *T*_0_. In Fig. 3B, the volcano plots for each screen shows the essential genes on the left side of the volcano plots (labeled as gene hits). The original study identified 1883 essential genes, whereas our new library screen identified 1817 essential genes (Fig. 3B). Additionally, a precision-recall analysis was performed using the Bayesian Analysis of Gene Essentiality 2 (BAGEL2) [23, 24] to determine the discrimination of essential genes for each library (Fig. 3C). The original study had an area under of the curve (AUC) of 0.920 and our new library had an AUC of 0.937 in the precision-recall plot. Between the 1000-fold screens, there is an overlap of 1366 essential genes (Fig. 3D). Some discrepancies in essential genes identified by each screen were expected due to differences in user handling, reagents, and cell doublings (~ 8 doublings in our screen compared to ~ 10).

There was greater overlap between our 1000 and 100-fold screens (Fig. 3D). This is expected since the same library and cell doublings were used compared to the legacy screen. Additionally, the legacy screen was performed years earlier in a different lab and parental K562 line. The 100-fold screen identified 1489 essential genes (Fig. 3B). The 100-fold screen had an AUC of 0.949 for the precision-recall analysis performed on essential genes identified, making the 100 × screening quality equivalent to the 1000 × screen (Fig. 3C). To compare the quality of the 100 and 1000-fold screens, we compared the phenotype scores for common gene hits. The pairwise comparison plot of the phenotype scores for gene hits shows a coefficient of determination of 0.900 (*r*^2^) using a linear least-squares regression (Fig. 3E). This demonstrates that our new library generates very similar gene-level results between 1000 and 100-fold coverage survival screens. While there was not perfect overlap between all three screens, the unique hits in each screen tended to fall near the cutoff populated by weaker and/or less significant hits (labeled in orange in Additional file 1: Fig. S7A). When plotting the phenotype score for unique gene hits in the legacy screen against the values in the LGR screen (Additional file 1: Fig. S7B), we see similar trends. 

**Fig. 4:**
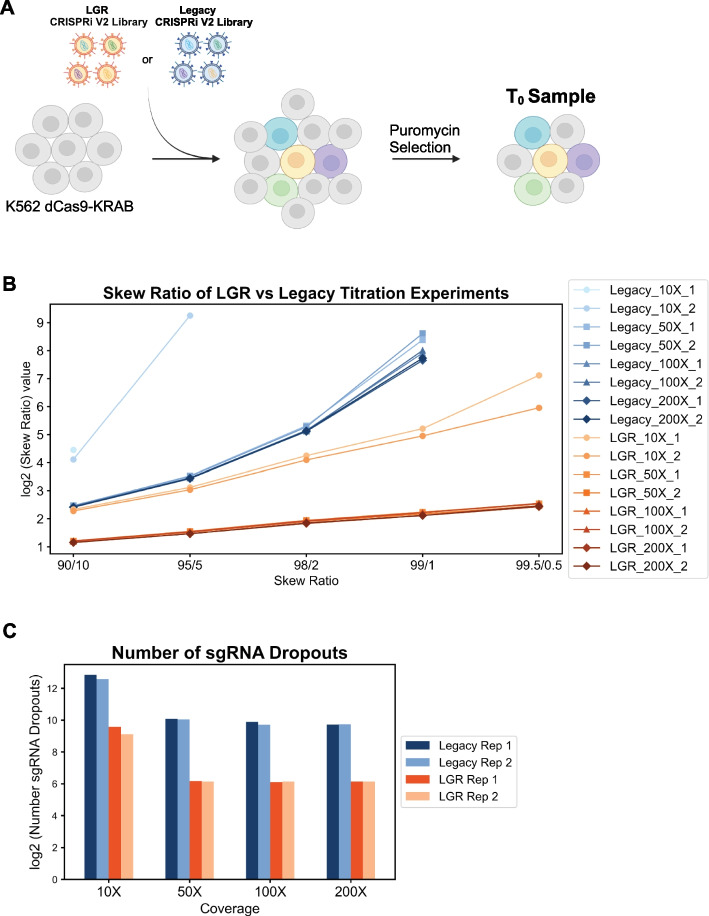
Transduction titration comparisons between the CRISPRi V2 LGR and the legacy libraries. **A** Schematic of the transduction experiments performed using the LGR and legacy libraries at 10, 50, 100, and 200-fold cell coverage. Each library at each coverage had a biological replicate. **B** Skew ratios for the LGR and legacy libraries transduced at 10, 50, 100, and 200-fold cell coverage. **C** sgRNA dropouts for the LGR and legacy libraries at 10, 50, 100, and 200-fold cell coverage

The strong correlation between the 100 and 1000-fold screens suggested we might be able to screen libraries at even lower coverages. To test this, we performed transductions at 200, 100, 50, and 10-fold coverage in technical duplicate using both the LGR and legacy libraries and examined guide dropouts and uniformity. The percent infection for these samples ranged from 11.5-22.8% and cells were treated with puromycin for 5 days until transduced cells accounted for approximately 90% of cells (Additional file 1: Fig. S6B). *T*_0_ samples were collected at this point and processed for NGS (Fig. 4A). The LGR library maintained lower skew ratios at all tested coverages (Fig. 4B, Additional file 1: Table S5). Furthermore, the 50 and 100-fold samples showed similar skew ratios to the 200-fold sample. In contrast, the legacy library skew ratio was worse at 100 and even worse at 50-fold (Fig. 4B, Additional file 1: Table S5). A major challenge running screens at lower cell coverage is guide RNA dropout. Our new library showed similar rates of guide dropouts between 200 down to 50-fold while the legacy library showed increased dropouts from 200 down to 50-fold (Fig. 4C, Additional file 1: Table S5). Furthermore, the number of guide dropouts with our library at 50-fold coverage is an order of magnitude less than the legacy library at 200-fold coverage. Notably, the majority (~ 94.62%) of sgRNA sequences that dropped out from our library began with a polyG sequence (Additional file 1: Table S6). These are due to a technical artifact of sequencing on the 2-color NextSeq 550 (see technical note in protocol). As expected, resequencing the plasmid libraries on the HiSeq 4000, a system not susceptible to polyG sequences at the beginning of the read resulted in dropout of only two guides and a lower skew ratio for our new CRISPRi library (Additional file 1: Table S7). This means the true number of dropouts in the 50-fold samples could be as low as 6**–**7 guides out of > 100,000 in the library. In contrast, sgRNA sequences that began with the polyG sequence only accounted for a minority of the dropouts in the legacy library (Additional file 1: Table S6). The low skew ratio and sgRNA dropout number for our library suggests this library can be used in screens with as little as 50-fold cell coverage, ~ 5 million cells for a 100,000-element library.

### Genome-wide CRISPR drug screen yields more hits with new library

**Fig. 5:**
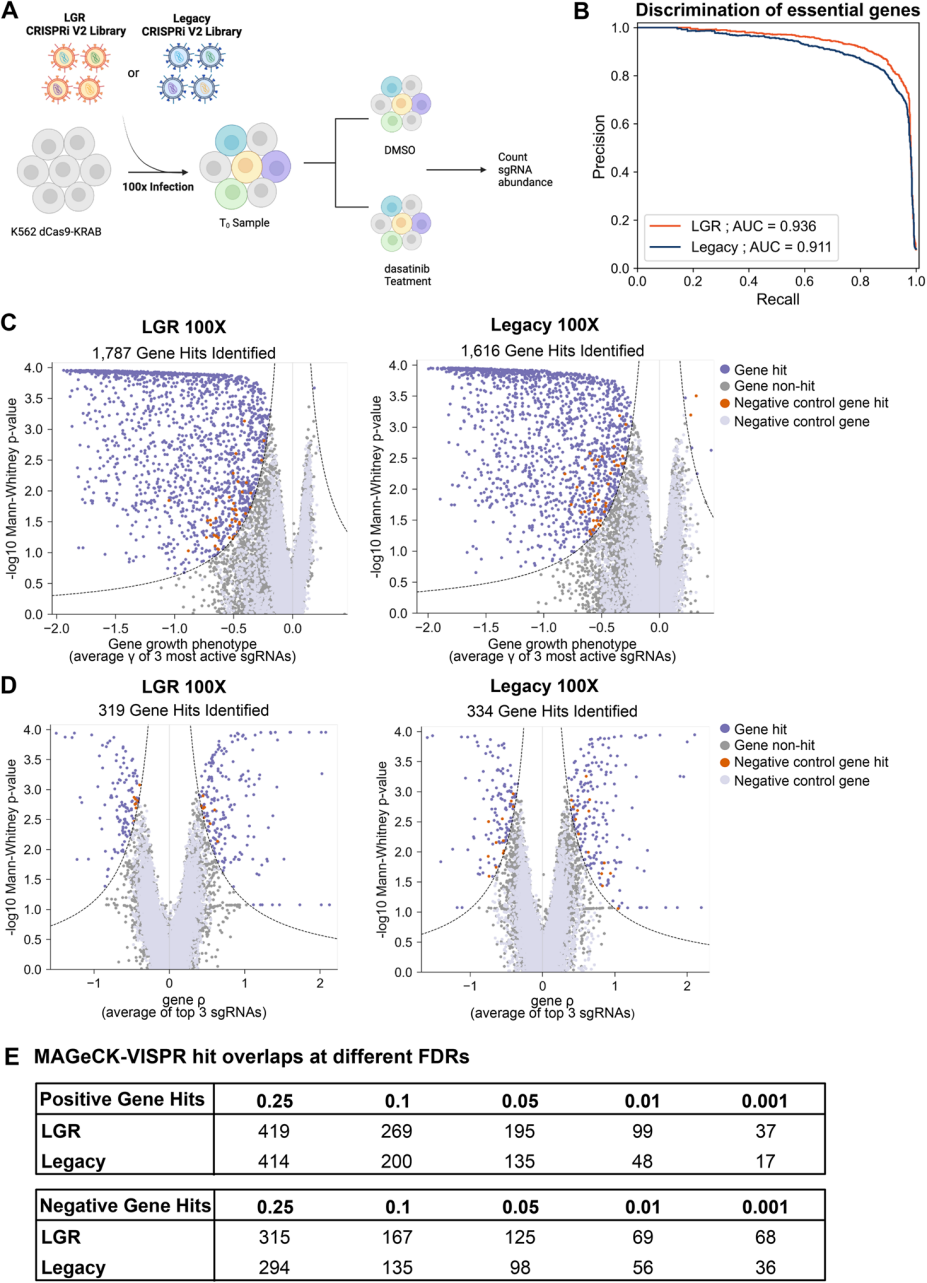
The CRISPRi V2 LGR library identifies more bonafide hits in a 100-fold cell coverage K562 dasatinib survival screen. **A** Schematic of the dasatinib survival screen performed using the LGR and legacy libraries at 100-fold cell coverage. **B** Precision-recall analysis of the essential genes identified in each library using BAGEL2 [23, 24] to measure screening quality. The area under the curve (AUC) for each library were as followed: LGR 0.936 and legacy 0.911. **C** Comparison of essential gene hits (*T*_0_ versus DMSO samples) identified in the LGR (right) versus the legacy (left) libraries. **D** Comparison of dasatinib treatment hits (dasatinib treatment versus DMSO control samples) identified in the LGR (right) versus the legacy (left) libraries. **E** MAGeCK-VISPR was used to determine the number of gene hits identified in each library at false discovery rates (FDR) ranging from 0.25 to 0.001. Hits were categorized as positive (increased survival) or negative (decreased survival)

To determine whether a high-quality screen can be performed at low coverage, we performed a drug survival screen on K562s, a chronic myeloid leukemia (CML) cell line, using dasatinib at 100-fold coverage (Fig. 5A). Dasatinib is a broad-spectrum tyrosine kinase inhibitor (TKI) that is approved for the treatment of CML [27–31]. However, there is no cure for CML as 40% of cases of clinical TKI failure occur in the setting of sustained BCR-ABL1 inhibition [32]. Identification of genes and biological pathways that are synthetically lethal in the context of CML treated with dasatinib or other TKIs could result in new CML therapies [33]. Given the performance of the LGR library at lower coverages, we hypothesized that a 100-fold coverage screen could be performed to comprehensively identify genes related to dasatinib resistance and sensitivity and demonstrate that lower coverage screens in other disease-relevant or otherwise complex systems could be performed with our improved library.

The dasatinib survival screens were performed at 100-fold cell coverage for both CRISPRi libraries (LGR and legacy) in parallel (Fig. 5A, Additional file 1: Fig. S6C). Each library was transduced once in K562s and then split to create technical replicates. The LGR and legacy libraries had 20% and 18% infection levels, respectively. The samples were treated with puromycin for 5 days (until approximately 90% enrichment), and 7 days after infection, the *T*_0_ samples were collected for each library. Each library had four technical replicates, two treated with 0.75 nM of dasatinib (single dose) and two treated with 0.01% DMSO (vehicle control) for 72 h (Additional file 1: Fig. S6C). After 72 h, the samples were grown in media without drug for 6 days. Dasatinib reduced cell viability as expected and after removal of drug, culture recovery was similar between the treated samples (Additional file 1: Fig. S8A). The LGR and legacy library samples (*T*_0_, DMSO, and dasatinib) clustered by library in the quality control heat map (Additional file 1: Fig. S8B). The PCA plots showed a separation of the libraries in PC1 (due to differences in guide abundance between the two libraries) whereas PC2 and PC3 were driven by biological conditions (Additional file 1: Fig. S8C). PC2 illustrates the effect of cell growth for 8 days after *T*_0_ and PC3 reflects the dasatinib treatment (Additional file 1: Fig. S8C). Treating the DMSO vehicle as the control arm in the survival screen, essential genes were identified for each library and a precision-recall analysis was performed using BAGEL2 [23, 24] to compare the discrimination of essential genes between the libraries (Fig. 5B). The LGR library had an AUC of 0.936 and the legacy library had an AUC of 0.911. In addition to having a higher quality library, more essential genes were identified with the LGR library (1787) than the legacy library (1616) (Fig. 5C). Dasatinib-specific gene hits were identified by ScreenProcessing (scored as rho), which represents the growth enrichment between the treated sample (dasatinib) and the untreated sample (DMSO) (Fig. 5D). Similar numbers of genes were identified by both libraries. Next, we used MAGeCK-VISPR [34, 35] to identify screen hits at various FDRs (Fig. 5E). At the highest FDR of 0.25, the legacy and LGR libraries had similar numbers of total gene hits (708 and 734, respectively). However, as stringency increased, the LGR library yielded more gene hits for both positive (increased cell survival) and negative (decreased cell survival) categories. At the most stringent FDR used (0.001), the LGR CRISPRi library had a total of 105 gene hits whereas the legacy library had 53.

The dasatinib gene hits for each library at FDRs of 0.25 and 0.001 were analyzed across the Gene Ontology (GO) and Kyoto Encyclopedia of Genes and Genomes (KEGG) databases to evaluate common biological pathways based on the hits generated [36–39]. The mediator complex (GO ID: 0016592) and the oxidative phosphorylation pathway (KEGG ID: hsa00190) were identified among the most significantly enriched annotations for the hit lists from both libraries (Additional file 1: Fig. S9). The mediator complex and the oxidative phosphorylation pathway have been shown to be potential drug targets to synergize with tyrosine kinase inhibitor treatments in CML. TKIs primarily target differentiated cells and fail to eliminate leukemic stem cells (LSCs) [40, 41]. However, inhibiting mitochondrial oxidative phosphorylation in combination with TKI treatment eliminates LSCs [42]. In a genome-wide CRISPR knockout screen, disruption of components of the mediator complex provided resistance against TKI treatment [43].

**Fig. 6:**
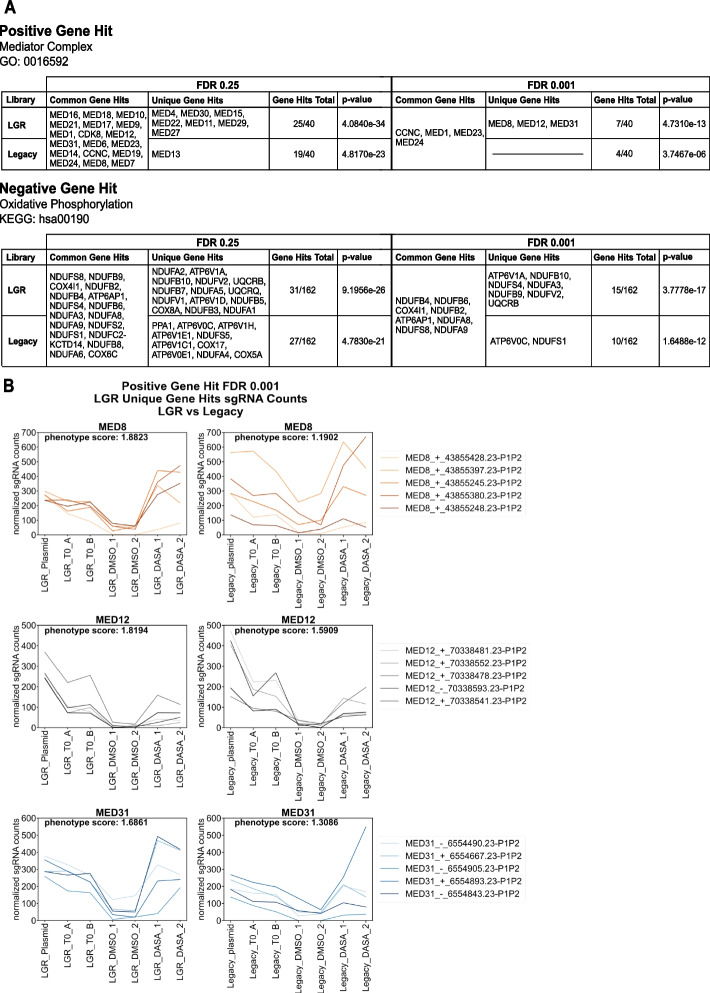
The top positive and negative gene hits in the dasatinib survival screens were investigated at 0.25 and 0.001 FDRs. **A** The list of gene hits determined by the LGR and legacy libraries for the Mediator Complex (top) and Oxidative Phosphorylation pathway (bottom). The Mediator Complex has a total of 40 genes associated with the cellular component and the Oxidative Phosphorylation pathway has 162. **B** Line plots of the normalized sgRNA counts for the LGR unique gene hits MED8, MED12, and MED31 for the LGR (left-side panel) and legacy (right-side panel) library samples at an FDR of 0.001. The gene phenotype score determined by MAGeCK is annotated on each subplot in the upper left-hand corner

In agreement with these two studies, we observe depletion of guides targeting the components of the oxidative phosphorylation pathway and enrichment of guides targeting the mediator complex in the dasatinib screen. Our new library generated more hits for the mediator complex and the oxidative phosphorylation pathway at an FDR of 0.25 and 0.001 (Fig. 6A). Additionally, the *p*-values of enrichment in both gene categories at both FDRs were markedly lower for the LGR library. Of the 40 genes annotated to be part of the mediator complex in the GO database, the screen with our library identified 7 of those genes (including all 4 of the legacy library hits) at an FDR of 0.001 (Fig. 6A). Additionally, our 100 × screen identified a similar number of components of the mediator complex (7 vs 10) to the previous CRISPR knockout screen performed at 250 × coverage [43]. Examining the individual guide abundance in the unique hits (Fig. 6B), we observe that silencing mediator components results in a growth defect and bottleneck for these guides. However, knockdown of these components promotes resistance to dasatinib treatment. The uniform abundance of individual guides in the LGR screen results in less variability after the treatment bottleneck. The screen sensitivity is improved because the read count differences between guides for a given gene can be more confidently identified as significant when there are fewer outliers and dropouts. Although the prior study used a CRISPR knockout library, several studies have shown strong correlation between CRISPR knockout and CRISPRi screens [44, 45]. When data from the previous study was filtered with the same 0.001 FDR with our screen, only one hit passed the cutoff. This further demonstrates the increased quality of screens performed with a less biased library. Additionally, another reason for differences between the CRISPRn screen and our CRISPRi-based results could be due to much higher rates of chromosome loss in CRISPRn editing [46] that can cause confounding effects in screens [47]. The combination of our survival screen, transduction titration experiments, and drug perturbation screen demonstrate the feasibility of screening at much lower cell coverages with our improved library.

## Discussion

In this work, we demonstrate that genome-wide CRISPR screens can be performed at smaller scale if high-quality libraries with high uniformity are used. We have developed an improved guide library cloning method that can be applied to applications beyond CRISPR that include any library cloned from oligo pools. This includes, but is not limited to shRNA, peptide, and barcode libraries. Through a combination of using forward and reverse oligo templates, optimizing insert amplification, and minimizing temperature during insert preparation steps, we have generated very uniform libraries that allow lower cell coverage screens. This has several practical benefits for screening.

First, starting with the same number of cells and same library size, one can screen 10–20 times more samples. These could be technical replicates, biological replicates, additional perturbations, additional cell lines or clones, or isogenic controls [48]. For example, while only two technical replicates were compared in analysis outlined for the 100 × survival screen, we had four replicates, which used 5-fold less cells than the duplicate 1000 × screen. Including all four replicates resulted in more essential genes hits (Additional file 1: Fig. S10A). These additional hits are likely real because the precision-recall AUC values were indistinguishable (two replicates = 0.949; four replicates = 0.945) (Additional file 1: Fig. S10B). Second, if the same number of cells are used, a 2 million element library could be screened instead of a library containing 100,000 elements. This enables experiments with larger libraries such as tiling screens to identify regulatory regions in non-coding sequences or synthetic combinatorial libraries. Third, due to the large number of cells that must be maintained in higher coverage screens, researchers often must split cells every day for several weeks. With lower cell coverage, cultures can be passaged at lower density while still maintaining adequate coverage and split every 2 or 3 days (Additional file 1: Table S8). Fourth, the majority of CRISPR screens have been performed in transformed cell lines because their cultures can easily be scaled up. This has been adequate for certain areas of biology such as cancer research, but many other interesting screening models such as differentiated iPSC cells, primary tissues, and difficult to transduce cells have been challenging to approach with genome-wide CRISPR screens [7, 8, 49]. The optimized CRISPRi and CRISPRa libraries described in this work provide a resource that make these models more tractable for genetic screens. Lastly, new compact dual-guide libraries [50] have reduced the number of elements to as few as one per gene. This allows lower usage of cells in single-gene screens as well as the ability to perform combinatorial screens. However, this precludes the calculation of *p*-values to filter hits. In single gene screens with our library at 50 × cell coverage minimizes the number of cells to similar levels as the dual-guide library, especially when factoring in the number of cells transduced with recombined lentiviral particles rates (~ 30%) of the dual guide systems.

## Conclusion

The optimizations developed here can be used to clone even more compact libraries, such as multi-guide Cas9 and Cas12a sgRNA constructs [50, 51]. With these multi-guide libraries, it is conceivable to have a guide library containing a single element per gene. A 20,000-guide library at 50-fold cell coverage only requires 1 million cells which can be propagated in a single 100-mm dish or multi-well plate. With automation, this can enable genome-wide guide screens of large panels of drugs or cellular genetic backgrounds, something unconceivable with existing libraries.

## Methods

### Cell lines

K562 cells acquired from the European Collection of Authenticated Cell Cultures (ECCAC) were cultured according to standard protocols and transfected with lentivirus containing the dCas9-KRAB construct. Cells were then sorted using a BD FACS Aria based off BFP signal. The pooled cell line was utilized for the essential gene drop-out screens, transduction experiments, and dasatinib screens. Cell lines were maintained in shaking cultures at 100 rpm at a concentration of 500,000 cells/mL for experiments. The parental cell line was authenticated by STR profiling.

### Plasmid vectors

The lentiviral expression vectors used in pooled CRISPR screen experimentations are available on Addgene under the following name: pLGR1002 (188320).

### LGR guide library cloning

A detailed protocol is in the supplemental materials (Additional file 5). Key points include synthesizing insert oligo pools in both forward and reverse complement orientations, minimizing over amplification of the insert, and performing gel electrophoresis size selection and extraction at low temperatures. To confirm sgRNA library representation and distribution, the plasmid DNA was sequenced (see “NGS sample prep and sequencing”).

### Plasmid library virus production and titers

Guide library virus was prepared using protocols from the Weissman Lab [52] with Lenti-X 293 T (Takara Bio, 632180). Virus titers were performed with polybrene (Millipore Sigma, TR-1003-G) in the K562 cell lines. Multiplicity of infection (MOI) and percent infection were determined by BFP signal using flow cytometry.

### Essential gene drop-out screens

K562 cells containing dCas9 machinery were infected at ~ 25%, targeting a 100- or 1000-fold cell coverage. After 2 days, transductants were selected with puromycin for 3 days until approximately 90% of the culture was BFP positive. The cultures were maintained at 500,000 cells/mL density for 7 days. Cell pellets were collected and frozen before DNA extraction. A schematic of the screen timeline is illustrated in Additional file 1: Fig. S6A.

### Dasatinib screens

Dasatinib (Millipore Sigma, SML2589) was diluted with DMSO to final concentration of 4 µM. Drug dosage for K562s was determined by performing a drug titration and a cell viability curve was generated. Approximately 10 million K562s with the dCas9-KRAB constructs were infected with either the LGR or legacy CRISPRi V2 libraries containing the top 5 guides (resulting in approximately 100-fold cell coverage). Puromycin treatment lasted for 5 days (until approximately 90% enrichment). Seven days after infection, the T_0_ samples were collected for each library. A single dose of 0.75 nM of dasatinib (determined from the titration experiment) was used as the drug selective pressure and 0.01% DMSO was used as a vehicle control. Treatment for 72 h was followed by 6 days of recovery. A schematic of the screen timeline is illustrated in Additional file 1: Fig. S6C. The LGR and legacy screens experienced similar cell death and recovery growth (Additional file 1: Fig. S8A).

### Genomic DNA processing

Genomic DNA was extracted using Macherey–Nagel Nucleospin Blood kits. The 10 × cell pellets were processed with the Mini kit (740951), the 50 × and 100 × pellets with the L kit (740954), and the 200 × and 1000 × pellets with the XL kit (740950). All pellets were processed according to the kit-specific protocols and quantified by Nanodrop.

### NGS sample prep and sequencing

Oligo pool NGS libraries were constructed with the Claret Bioscience SRSLY PicoPlus kit (K250B-24) according to manufacturer instructions with 20–25 ng oligo template and PCR amplification using the NEBNext Ultra II Q5 Master Mix (M0544). Oligo pool NGS libraries were prepared using 10 PCR cycles and an 8-bp dual index primer set with the sequence AATGATACGGCGACCACCGAGATCTACACnnnnnnnnACACTCTTTCCCTACACGACGCTCTTCCGATCT and CAAGCAGAAGACGGCATACGAGATnnnnnnnnGTGACTGGAGTTCAGACGTGTGCTCTTCCGATCT using the following PCR conditions: denaturation at 98˚C for 30 s, 10 cycles of denaturation at 98˚C for 10 s, annealing and extension at 65˚C for 75 s, and final extension at 65˚C for 5 min. The indexed libraries were purified with the 1 × of the DNA purification magnetic beads (Omega Biotek: Mag-Bind® Total Pure NGS, M1378) and eluted with the TE buffer. The oligo pool libraries were pooled and sequenced on a NextSeq 550 with 90 cycles.

Following DNA extraction of cell pellets, screening samples were prepared for NGS sequencing by a single PCR amplification; 24 cycles of PCR were performed using NEBNext Ultra II Q5 Master Mix (M0544). PCR primer sequences are provided in the supplemental information (Additional file 6). The forward primers (5' PCR primers) used in the NGS library preparation for gDNA samples contained a 6-bp index sample barcode: AATGATACGGCGACCACCGAGATCTACACGATCGGAAGAGCACACGTCTGAACTCCAGTCACnnnnnnGCACAAAAGGAAACTCACCCT. For sgRNA libraries cloned into the pLGR1002 vector, the following reverse primer was used:

CAAGCAGAAGACGGCATACGAGATATGCTGTTTCCAGCTTAGCTCTT. Legacy libraries used the following reverse primer: CAAGCAGAAGACGGCATACGAGATCGACTCGGTGCCACTTTTTC. Due to different *T*_m_ of the reverse primers, the LGR PCR samples were amplified with an annealing temperature of 62.6˚C, while the legacy samples were amplified with an annealing temperature of 65˚C. All PCRs were conducted in 100 µL volumes with 10 µg of DNA per reaction. All PCRs had the following cycling conditions: a hot start at 98˚C for 30 s, 24 cycles of denaturation at 98˚C for 10 s then annealing for 75 s at the appropriate annealing temperature, a final elongation at 65˚C for 5 min, with a final 4˚C hold. After PCR, aliquots of each set of reactions were pooled. The legacy samples were purified using 0.65 × and then 1 × doubled-sided SPRI beads while the LGR samples using 0.65 × and then 1.2 × double-sided SPRI beads. The purified and pooled LGR and legacy samples were quantified on the TapeStation using the High Sensitivity Kit before being sequenced on the NextSeq 550 with a custom sequencing primer (GTGTGTTTTGAGACTATAAGTATCCCTTGGAGAACCACCTTGTTGG) that was spiked in with the standard Illumina sequencing primer (6 µL of the 100 µM custom sequencing primer was added). PhiX control library was spiked in at 10% PhiX to increase base diversity for the single-end 20 cycles sequencing runs. Note that the first several cycles of sequencing on the NextSeq 550 are used to identify clusters. Because G-bases are dark in two-color sequencing systems, guides that contain begin with a polyG sequence are difficult or impossible to identify. This issue will also be present on the MiniSeq platform. Two-colored patterned flow cell systems such as the NovaSeq and NextSeq 1000/2000 should not be affected by this. An alternative method to address this issue with the NextSeq 500/550 is to use a staggered sequencing approach.

### Computational analysis of screens

Read counts were processed using an iteration of ScreenProcessing (https://github.com/ucsf-lgr/ScreenProcessing) developed by the Weissman Lab. ScreenProcessing [5, 53] analyzes pooled CRISPR screens by comparing the sgRNAs targeting each gene of interest with the entire set of sgRNAs targeting all genes. sgRNAs are ranked according to their enrichment score, which is the comparison of phenotype distributions of sgRNAs targeting each gene of interest with the non-targeting control (NTC) sgRNAs that are not predicted to bind the genome [18]. The NTCs serve as a reliable null distribution. Genes are ranked according to the phenotype scores and *p*-values derived from the sgRNA rankings. The gene phenotype score is the average of the absolute value of log_2_ enrichment score of the top 3 sgRNAs targeting the gene [53]. The *p*-value for a gene is calculated using the Mann–Whitney *U* test (MW test) by comparing all 5 sgRNAs to the NTCs. A gene is considered a hit for a false discovery rate (FDR) of < 0.05. The gene scores are visualized in a volcano plot, where the phenotype effect size is on the *x*-axis and the *p*-value is on the *y*-axis. Statistical precision and recall of essential and non-essential genes set for libraries were calculated for genes ranked by growth phenotype using BAGEL2 [24]. The area under the curve (AUC) values were calculated using scikit-learn [54].

Quality control plots as well as gene hit analysis were performed with MAGeCK-Vispr [34, 35]. MAGeCK-Vispr uses the negative binomial *p*-value to perform sgRNA ranking and the expectation maximization (EM) algorithm to perform gene ranking with adjustable FDR cutoffs. Gene Onology (GO) and KEGG pathway analysis for the dasatinib screens were performed with clusterProfiler [25, 26, 55, 56] and DAVID [57, 58].

### Supplementary Information

**Additional file 1**

**Additional file 2**

**Additional file 3**

**Additional file 4.**

**Additional file 5.**

**Additional file 6.**

**Additional file 7.**

**Additional file 8.**

**Additional file 9.**

**Additional file 10.**

**Additional file 11.**

**Additional file 12.**

**Additional file 13.**

**Additional file 14.**

**Additional file 15.**

## Data Availability

All data generated or analyzed during this study are included in this published article (and its supplementary information files). ScreenProcessing is an open source bioinformatics pipeline available in the LGR’s GitHub repository (https://github.com/ucsf-lgr/ScreenProcessing) that was developed by Horlbeck et. al. (2016) and is also available at (https://github.com/mhorlbeck/ScreenProcessing) [5, 18, 48]. MAGeCK-Vispr is an open source pipeline developed by Wei Lei and Han Xu from the Dr.Xiaole Shirley Liu laboratory and is available on SOURCEFORCE (https://sourceforge.net/p/mageck/wiki/Home/) [34, 35]. BAGEL2 is an open source pipeline developed by the Hart Lab available in the Hart Lab’s GitHub repository (https://github.com/hart-lab/bagel) [24]. ClusterProfiler is an open source enrichment tool analysis R package available on Bioconductor (https://doi.org/10.18129/B9.bioc.clusterProfiler) [25, 26, 55, 56]. The Database for Annotation Visualization and Integrated Discovery (DAVID) is an open source functional annotation tool that is available online (https://david.ncifcrf.gov/home.jsp) [57, 58]. Raw NGS count files used for the analysis of skew ratios for publicly available CRISPR libraries are listed in Additional file 4. FASTQ sequences generated from this study have been deposited with the Gene Expression Omnibus (GEO) under the accession number GSE222531 [59].
