# Supplementary material for: Compact CRISPR genetic screens enabled by improved guide RNA library cloning
Source: Genome Biol. 2024 Jan 19;25:25. doi: 10.1186/s13059-023-03132-3 (PMC10797759; doi:10.1186/s13059-023-03132-3)
Supplement: Supplementary file 1 — Additional file 1 [file 13059_2023_3132_MOESM1_ESM.pdf]

## Fig. S1

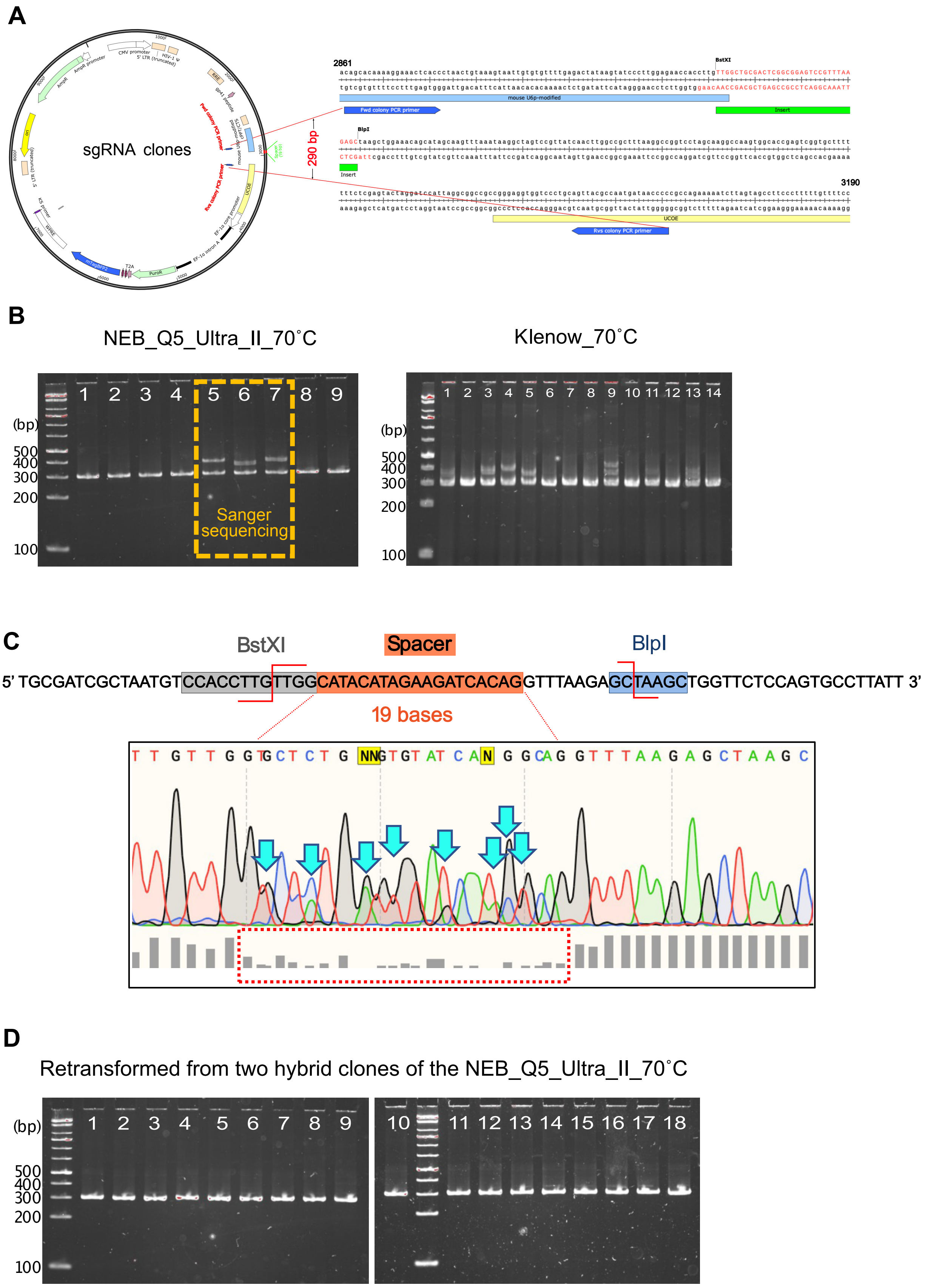
A
sgRNA clones
290 bp
2861
3190
BstXI
BlpI
mouse U6p-modified
Fwd colony PCR primer
Insert
UCOE
Rvs colony PCR primer
B
NEB_Q5_Ultra_II_70˚C
Klenow_70˚C
(bp)
500
400
300
200
100
Sanger
sequencing
C
BstXI
Spacer
BlpI
5' TGCGATCGCTAATGTCCACCTTGTTGGCATACATAGAAGATCACAGGTTTAAGAGCTAAGCTGGTTCTCCAGTGCCTTATT 3'
19 bases
T T G T T G G T G C T C T G NN G T G T A T C A N G G C A G G T T T A A G A G C T A A G C
D
Retransformed from two hybrid clones of the NEB_Q5_Ultra_II_70˚C

Fig S1. (A) Diagram of the cloning strategy and colony PCR primer design. (B) Colony PCR of sgRNA clones and gel electrophoresis. (C) Representative Sanger sequencing trace of plasmids from hybrid clones (D) Plasmids from two different hybrid clones were retransformed. Colony PCR was performed, and hybrid clones were not detected.

## Fig. S2

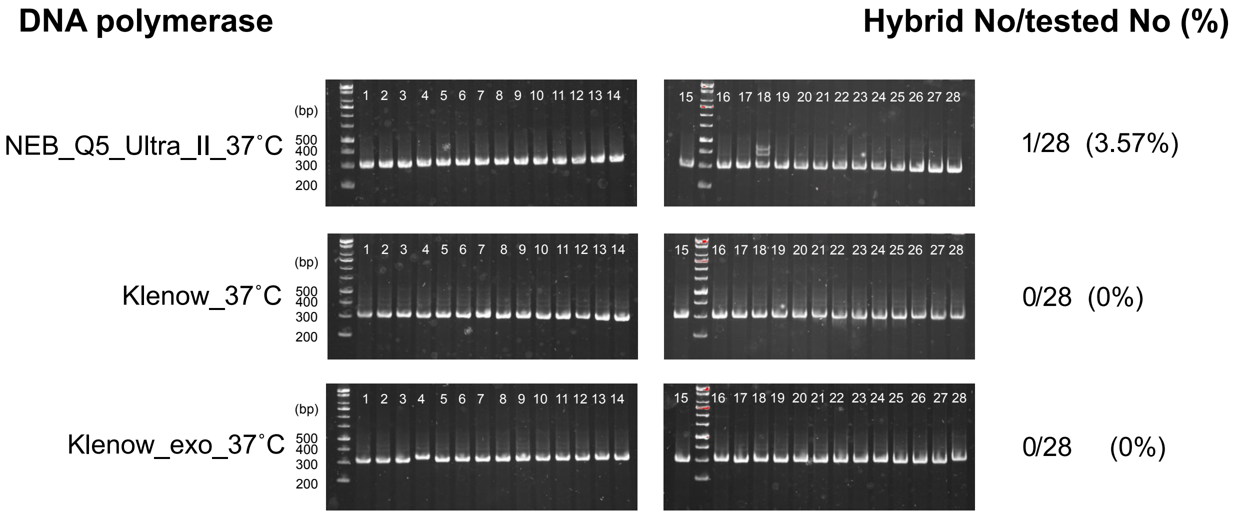


Fig S2. Lowering the elution temperature for the insert gel extraction reduces the formation of hybrid clones, regardless of the insert preparation polymerase used.

## Fig. S3

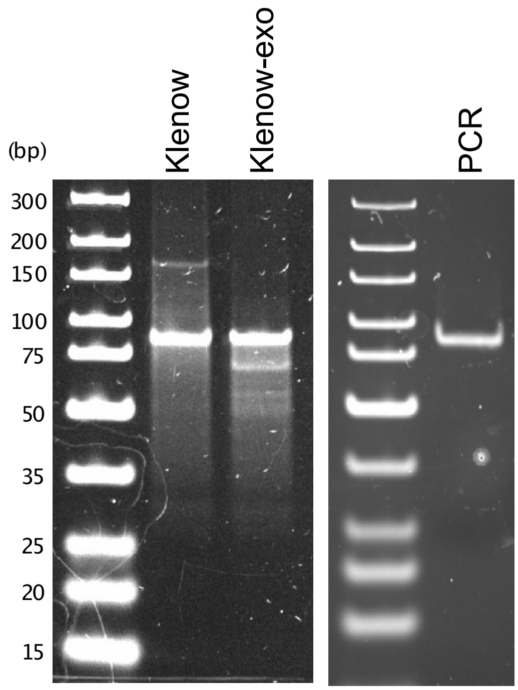


Fig S3. Synthesis of double-stranded sgRNA with the three different DNA polymerases (Klenow, Klenow exo-, and NEB Q5 Ultra II) and gel electrophoresis indicates less by-products with PCR.

**Fig. S4**

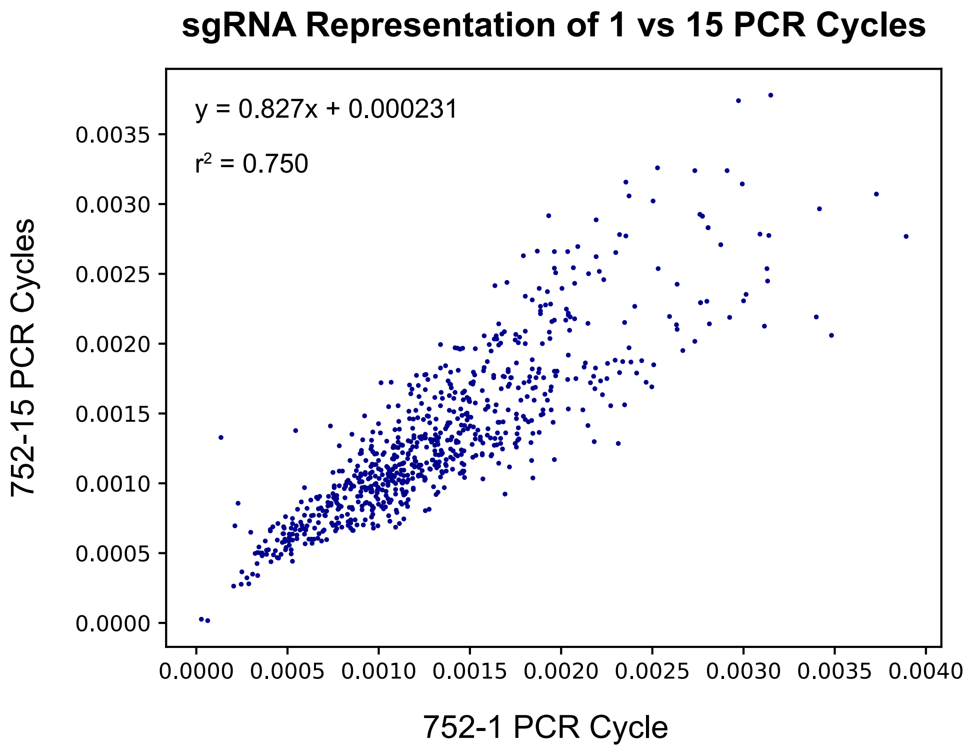


Fig S4. Additional PCR cycles introduce minimal bias in sgRNA libraries. An oligo pool containing 752 guides was amplified by one or 15 cycles of PCR, digested, and cloned into a guide vector. A pairwise comparison of sequence counts of guides in each library is depicted and a linear least-squares regression was performed.

**Fig. S5**

**A**

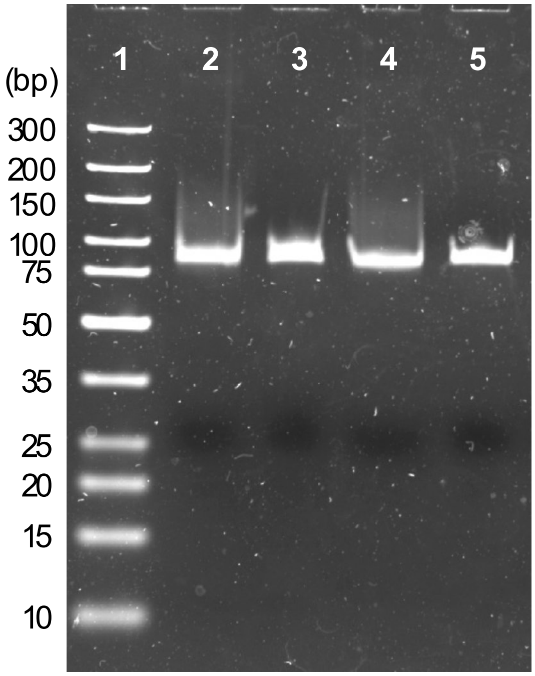
1 2 3 4 5
(bp)
300
200
150
100
75
50
35
25
20
15
10

**B**

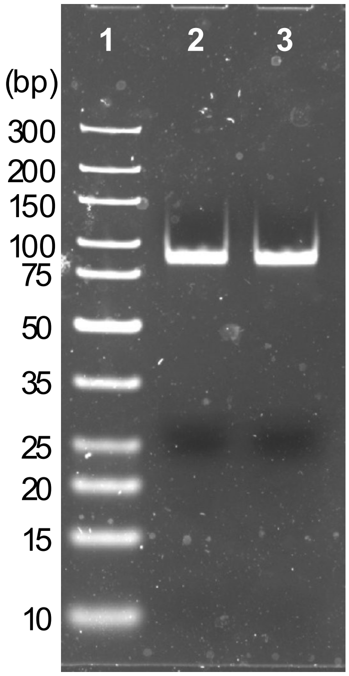
1 2 3
(bp)
300
200
150
100
75
50
35
25
20
15
10

Fig S5. Optimization of PCR cycles and input template concentration reduces overamplification products. (A) Lane 1: DNA ladder, Lanes 2 and 4: 200pM template. Lanes 3 and 5: 10pM template. Lanes 2-5: 13 cycles PCR. (B) Lane 1: DNA ladder. Lane 2: 200pM template, Lane 3: 600pM template. Lanes 2 and 3: 8 cycles PCR.

**Table S1**

**CRISPRi - LGR**

| | # Guides | Skew 90/10 | Skew 95/5 | Skew 98/2 | Skew 99/1 | Skew 99.5/0.5 |
|---|---|---|---|---|---|---|
| **Entire Library** | 103,074 | 1.99 | 2.36 | 2.86 | 3.25 | 3.73 |
| **h1** | 12,930 | 2.00 | 2.37 | 2.86 | 3.28 | 3.67 |
| **h2** | 16,197 | 1.99 | 2.36 | 2.84 | 3.25 | 3.73 |
| **h3** | 16,699 | 1.99 | 2.39 | 2.89 | 3.26 | 3.71 |
| **h4** | 12,161 | 1.99 | 2.36 | 2.88 | 3.27 | 3.79 |
| **h5** | 12,509 | 1.99 | 2.36 | 2.87 | 3.28 | 3.75 |
| **h6** | 13,090 | 1.97 | 2.32 | 2.80 | 3.15 | 3.66 |
| **h7** | 19,488 | 1.97 | 2.33 | 2.81 | 3.22 | 3.72 |

Table S1. Skew ratios of the genome-wide CRISPRi LGR library broken down by sublibraries.

**Table S2**

**CRISPRi - Legacy**

| | # Guides | Skew 90/10 | Skew 95/5 | Skew 98/2 | Skew 99/1 | Skew 99.5/0.5 |
|---|---|---|---|---|---|---|
| **Entire Library** | 103,074 | 5.11 | 10.27 | 33.29 | 171.38 | inf |
| **h1** | 12,930 | 4.97 | 8.61 | 19.00 | 33.77 | 102.39 |
| **h2** | 16,197 | 3.27 | 5.03 | 9.87 | 17.60 | 36.77 |
| **h3** | 16,699 | 2.91 | 4.24 | 6.90 | 11.04 | 19.07 |
| **h4** | 12,161 | 3.24 | 4.91 | 8.46 | 13.88 | 27.36 |
| **h5** | 12,509 | 4.51 | 6.87 | 11.98 | 19.05 | 33.90 |
| **h6** | 13,090 | 8.78 | 21.25 | 130.06 | inf | inf |
| **h7** | 19,488 | 12.89 | 44.59 | inf | inf | inf |

Table S2. Skew ratios of the genome-wide CRISPRi Legacy library broken down by sublibraries.

## Table S3

**CRISPRa - LGR**

| | # Guides | Skew 90/10 | 95/5 | 98/2 | 99/1 | 99.5/0.5 |
|---|---|---|---|---|---|---|
| **Entire Library** | 101,250 | 1.98 | 2.46 | 3.17 | 3.91 | 5.10 |
| **h1** | 12,751 | 2.01 | 2.51 | 3.22 | 3.94 | 5.47 |
| **h2** | 15,918 | 2.01 | 2.51 | 3.20 | 3.99 | 5.26 |
| **h3** | 16,277 | 1.97 | 2.42 | 3.14 | 3.85 | 4.95 |
| **h4** | 11,909 | 1.99 | 2.49 | 3.27 | 4.15 | 5.61 |
| **h5** | 12,207 | 1.98 | 2.45 | 3.26 | 4.08 | 5.78 |
| **h6** | 12,906 | 1.96 | 2.41 | 3.07 | 3.66 | 4.58 |
| **h7** | 19,282 | 1.97 | 2.41 | 3.07 | 3.80 | 4.54 |

Table S3. Skew ratios of the genome-wide CRISPRa LGR library broken down by sublibraries.

## Table S4

**CRISPRa - Legacy**

| | # Guides | Skew 90/10 | 95/5 | 98/2 | 99/1 | 99.5/0.5 |
|---|---|---|---|---|---|---|
| **Entire Library** | 101,250 | 2.80 | 3.97 | 6.57 | 10.99 | 23.12 |
| **h1** | 12,751 | 3.04 | 4.33 | 7.11 | 11.42 | 19.97 |
| **h2** | 15,918 | 2.76 | 4.00 | 6.78 | 11.66 | 29.54 |
| **h3** | 16,277 | 2.74 | 4.12 | 7.65 | 14.83 | 32.68 |
| **h4** | 11,909 | 2.55 | 3.57 | 6.26 | 10.42 | 16.50 |
| **h5** | 12,207 | 2.90 | 4.13 | 6.63 | 10.61 | 53.46 |
| **h6** | 12,906 | 2.08 | 2.67 | 3.55 | 4.52 | 7.25 |
| **h7** | 19,282 | 2.77 | 3.95 | 6.86 | 11.65 | 22.00 |

Table S4. Skew ratios of the genome-wide CRISPRa Legacy library broken down by sublibraries.

# Fig. S6

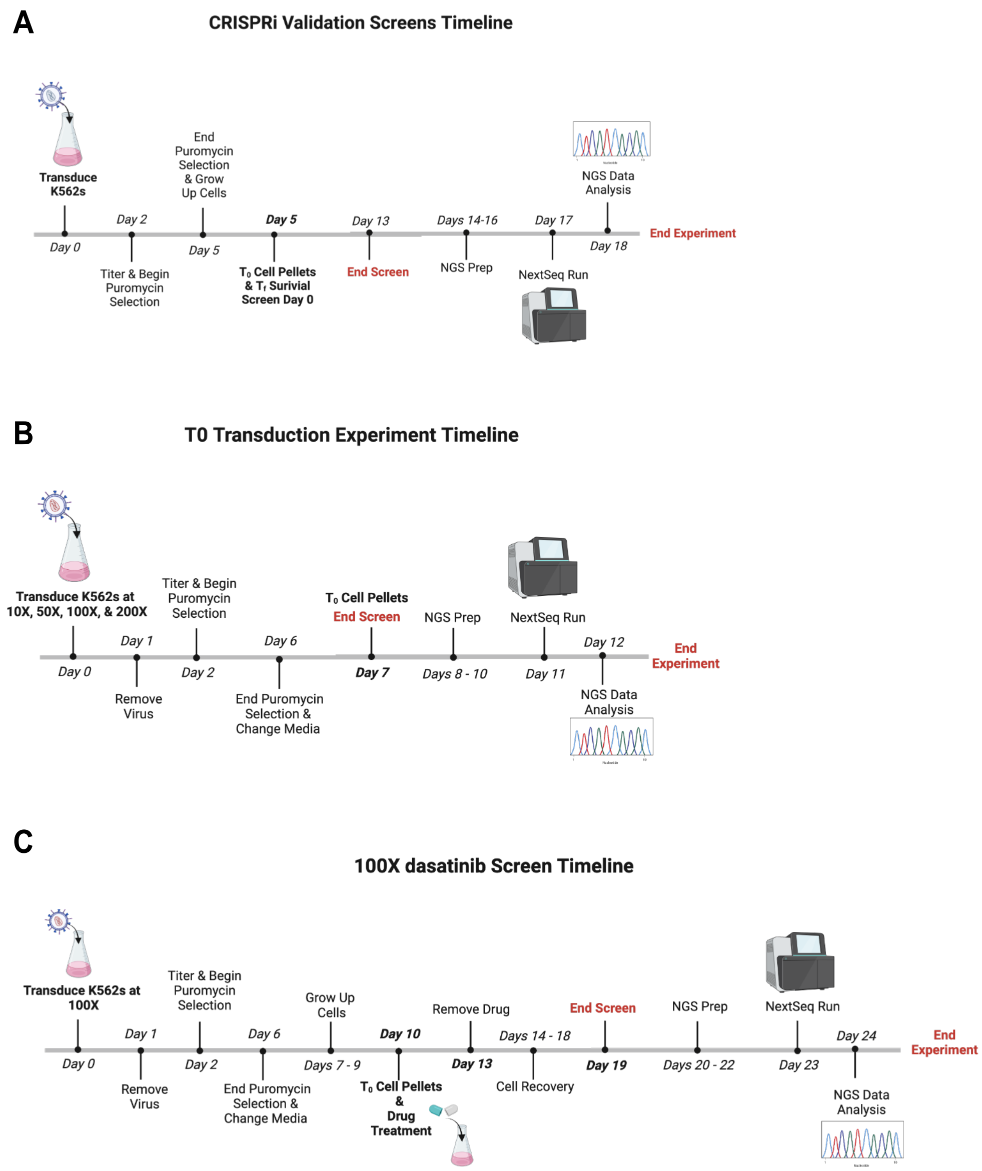


Fig S6. Schematic of the screening timelines for the CRISPRi V2 validation screen (Figure 3), the transduction titration experiment (Figure 4), and the dasatinib survival screen (Figure 5). Timelines include the transduction duration, virus titer determination, puromycin selection length, cell pellet collections, and next-generation sequencing (NGS) sample preparation for each experiment. (A) CRISPRi V2 LGR 1000 and 100-fold cell coverage survival screen timeline. (B) CRISPRi V2 LGR verses legacy transduction titration experiment timeline. (C) LGR verses legacy 100-fold cell coverage dasatinib survival screen timeline.

# Fig. S7

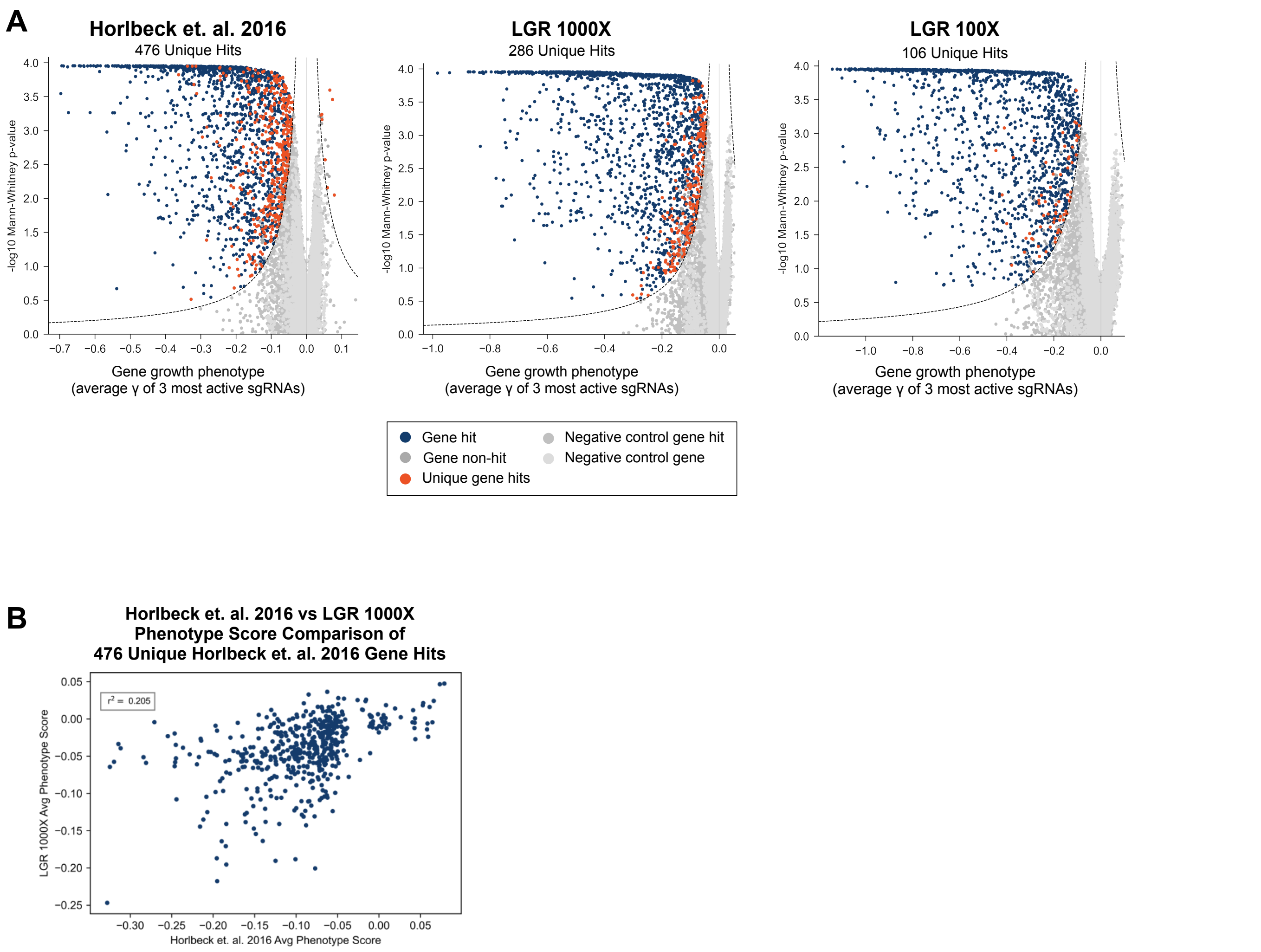


Fig S7. (A) Volcano plots of all three survival screens (Horlbeck et al. 2016, LGR 1000-fold cell coverage, and LGR 100-fold cell coverage). Unique gene hits identified for each library are orange and gene hits that are shared among the libraries are in blue. Unique gene hits for each library are near the cutoff (dotted lines) which are populated by weaker and/or less significant hits. (B) A point comparison of the phenotype score of the 476 unique gene hits found in the Horlbeck et al. 2016 survival screen between the Horlbeck et al. 2016 and LGR 1000-fold screens. The coefficient of determination ($r^2$) was calculated using a linear least-squares regression.

## Table S5

| Sample | 90/10 | 95/5 | 98/2 | 99/1 | 99.5/0.5 | Number sgRNA Dropouts |
|---|---|---|---|---|---|---|
| LGR_10X_1 | 5.04 | 8.7 | 19 | 37.06 | 138.8 | 765 |
| LGR_10X_2 | 4.85 | 8.2 | 17.07 | 30.95 | 62.17 | 553 |
| Legacy_10X_1 | 21.88 | – | – | – | – | 7409 |
| Legacy_10X_2 | 17.29 | 610 | – | – | – | 6141 |
| LGR_50X_1 | 2.31 | 2.93 | 3.84 | 4.72 | 5.81 | 72 |
| LGR_50X_2 | 2.28 | 2.86 | 3.73 | 4.5 | 5.51 | 71 |
| Legacy_50X_1 | 5.51 | 11.56 | 39.8 | 333.95 | – | 1092 |
| Legacy_50X_2 | 5.54 | 11.42 | 38.53 | 392 | – | 1056 |
| LGR_100X_1 | 2.29 | 2.91 | 3.82 | 4.67 | 5.77 | 69 |
| LGR_100X_2 | 2.31 | 2.89 | 3.78 | 4.65 | 5.86 | 71 |
| Legacy_100X_1 | 5.42 | 11 | 35.5 | 241.5 | – | 950 |
| Legacy_100X_2 | 5.45 | 10.97 | 34.69 | 257.5 | – | 841 |
| LGR_200X_1 | 2.22 | 2.76 | 3.56 | 4.33 | 5.39 | 71 |
| LGR_200X_2 | 2.22 | 2.76 | 3.6 | 4.34 | 5.48 | 71 |
| Legacy_200X_1 | 5.32 | 10.8 | 34.53 | 201.29 | – | 844 |
| Legacy_200X_2 | 5.35 | 10.91 | 35.06 | 212.67 | – | 860 |

Table S5. The majority of dropouts identified in the CRISPRi V2 LGR library (plasmid and K562 samples) was due to the Illumina NextSeq 550 sequencing artifact. The NextSeq 550 is a two-color sequencer that requires signal in the first few cycles to find clusters on the flow cell. Since G bases are dark and lack signal, sgRNAs that begin with a polyG are not detected. Guide library skew ratios and number of sgRNA dropouts in the LGR and legacy samples at 10, 50, 100, and 200-fold cell coverage.

## Table S6

| Samples | Total sgRNAs Drop Out | % Total Guides | % of Poly G Drop Outs |
|---|---|---|---|
| LGR_10X_1 | 765 | 0.742 | 8.627 |
| LGR_10X_2 | 553 | 0.537 | 11.754 |
| Legacy_10X_1 | 7409 | 7.188 | 1.039 |
| Legacy_10X_2 | 6141 | 5.958 | 1.238 |
| LGR_50X_1 | 72 | 0.070 | 90.278 |
| LGR_50X_2 | 71 | 0.069 | 90.141 |
| Legacy_50X_1 | 1092 | 1.059 | 5.861 |
| Legacy_50X_2 | 1056 | 1.025 | 6.155 |
| LGR_100X_1 | 69 | 0.067 | 94.203 |
| LGR_100X_2 | 71 | 0.069 | 92.958 |
| Legacy_100X_1 | 950 | 0.922 | 6.842 |
| Legacy_100X_2 | 841 | 0.816 | 7.729 |
| LGR_200X_1 | 71 | 0.069 | 88.732 |
| LGR_200X_2 | 71 | 0.069 | 88.732 |
| Legacy_200X_1 | 844 | 0.819 | 7.464 |
| Legacy_200X_2 | 860 | 0.834 | 7.093 |
| Plasmid LGR NextSeq | 59 | 0.057 | 93.220 |
| Plasmid LGR HiSeq | 2 | 0.002 | 0.000 |
| Plasmid Legacy NextSeq | 770 | 0.747 | 14.545 |

Table S6. In a 103,074 element library, the majority of guides that drop out of the LGR library samples at higher fold cell coverage (50, 100, and 200) are sgRNAs that begin with polyG sequences. Whereas the legacy library samples at the same cell coverages experienced dropouts due to the library quality.

## Table S7

| LGR CRISPRi Plasmid HiSeq | | LGR CRISPRi Plasmid NextSeq | |
|---|---|---|---|
| Skew : 1.92 | | Skew : 1.99 | |
| **Frequency of Drop Out** | **First 5 Bases of sgRNA** | **Frequency of Drop Out** | **First 5 Bases of sgRNA** |
| 1 | GTCAC | 56 | GGGGG |
| 1 | GCCCG | 1 | GTCAC |
| | | 1 | GGGGA |
| | | 1 | GGCTC |

Table S7. Sequence composition analysis of the CRISPRi V2 LGR plasmid library determined that most sgRNAs dropping out began with the polyG sequence in the NextSeq 550 run. When the same library was sequence on the four-color HiSeq 4000, much fewer guides dropped out and a lower 90/10 skew ratio was achieved.

**Fig. S8**

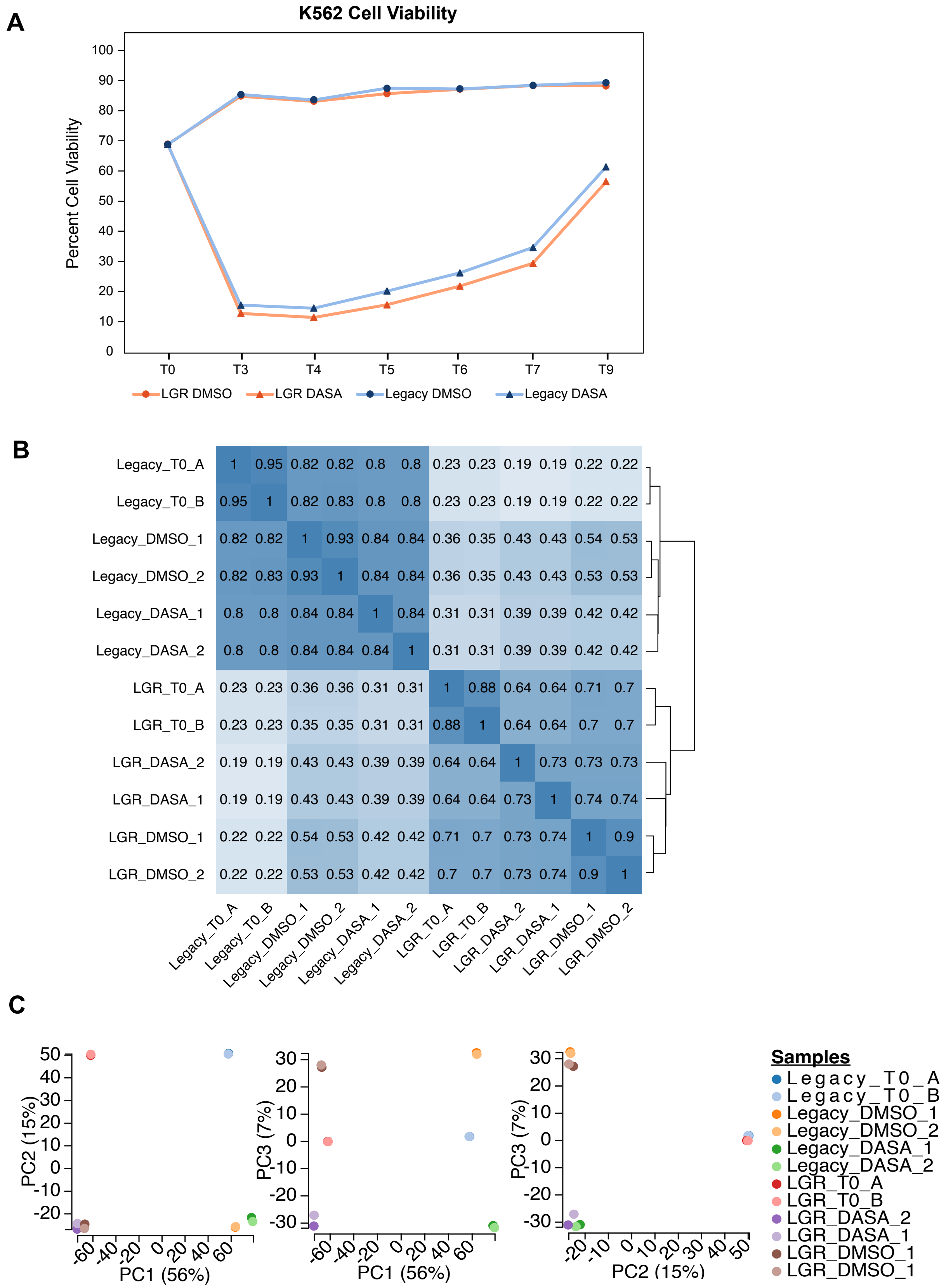
A
K562 Cell Viability
Percent Cell Viability
0
10
20
30
40
50
60
70
80
90
100
T0
T3
T4
T5
T6
T7
T9
LGR DMSO
LGR DASA
Legacy DMSO
Legacy DASA
B
Legacy_T0_A 1 0.95 0.82 0.82 0.8 0.8 0.23 0.23 0.19 0.19 0.22 0.22
Legacy_T0_B 0.95 1 0.82 0.83 0.8 0.8 0.23 0.23 0.19 0.19 0.22 0.22
Legacy_DMSO_1 0.82 0.82 1 0.93 0.84 0.84 0.36 0.35 0.43 0.43 0.54 0.53
Legacy_DMSO_2 0.82 0.83 0.93 1 0.84 0.84 0.36 0.35 0.43 0.43 0.53 0.53
Legacy_DASA_1 0.8 0.8 0.84 0.84 1 0.84 0.31 0.31 0.39 0.39 0.42 0.42
Legacy_DASA_2 0.8 0.8 0.84 0.84 0.84 1 0.31 0.31 0.39 0.39 0.42 0.42
LGR_T0_A 0.23 0.23 0.36 0.36 0.31 0.31 1 0.88 0.64 0.64 0.71 0.7
LGR_T0_B 0.23 0.23 0.35 0.35 0.31 0.31 0.88 1 0.64 0.64 0.7 0.7
LGR_DASA_2 0.19 0.19 0.43 0.43 0.39 0.39 0.64 0.64 1 0.73 0.73 0.73
LGR_DASA_1 0.19 0.19 0.43 0.43 0.39 0.39 0.64 0.64 0.73 1 0.74 0.74
LGR_DMSO_1 0.22 0.22 0.54 0.53 0.42 0.42 0.71 0.7 0.73 0.74 1 0.9
LGR_DMSO_2 0.22 0.22 0.53 0.53 0.42 0.42 0.7 0.7 0.73 0.74 0.9 1
Legacy_T0_A
Legacy_T0_B
Legacy_DMSO_1
Legacy_DMSO_2
Legacy_DASA_1
Legacy_DASA_2
LGR_T0_A
LGR_T0_B
LGR_DASA_2
LGR_DASA_1
LGR_DMSO_1
LGR_DMSO_2
C
PC2 (15%)
PC1 (56%)
PC3 (7%)
PC1 (56%)
PC3 (7%)
PC2 (15%)
Samples
Legacy_T0_A
Legacy_T0_B
Legacy_DMSO_1
Legacy_DMSO_2
Legacy_DASA_1
Legacy_DASA_2
LGR_T0_A
LGR_T0_B
LGR_DASA_2
LGR_DASA_1
LGR_DMSO_1
LGR_DMSO_1

Fig S8. Dasatinib survival screen quality control measurements for the LGR and legacy library samples. (A) K562 cell viability graph for the average LGR and legacy dasatinib treated (DASA) and control (DMSO) samples over the 10 days of the experiment. Dasatinib treatment was for 72 hours (T0 - T3) and recovery was for 5 days (T3 – T9). (B) Hierarchical clustering of the LGR and legacy library samples using Pearson correlation. (C) Principal Component Analysis (PCA) plot of the LGR and legacy library samples.

**Fig. S9**

## Top 12 Barplots of GO & KEGG annotations
## FDR 0.001

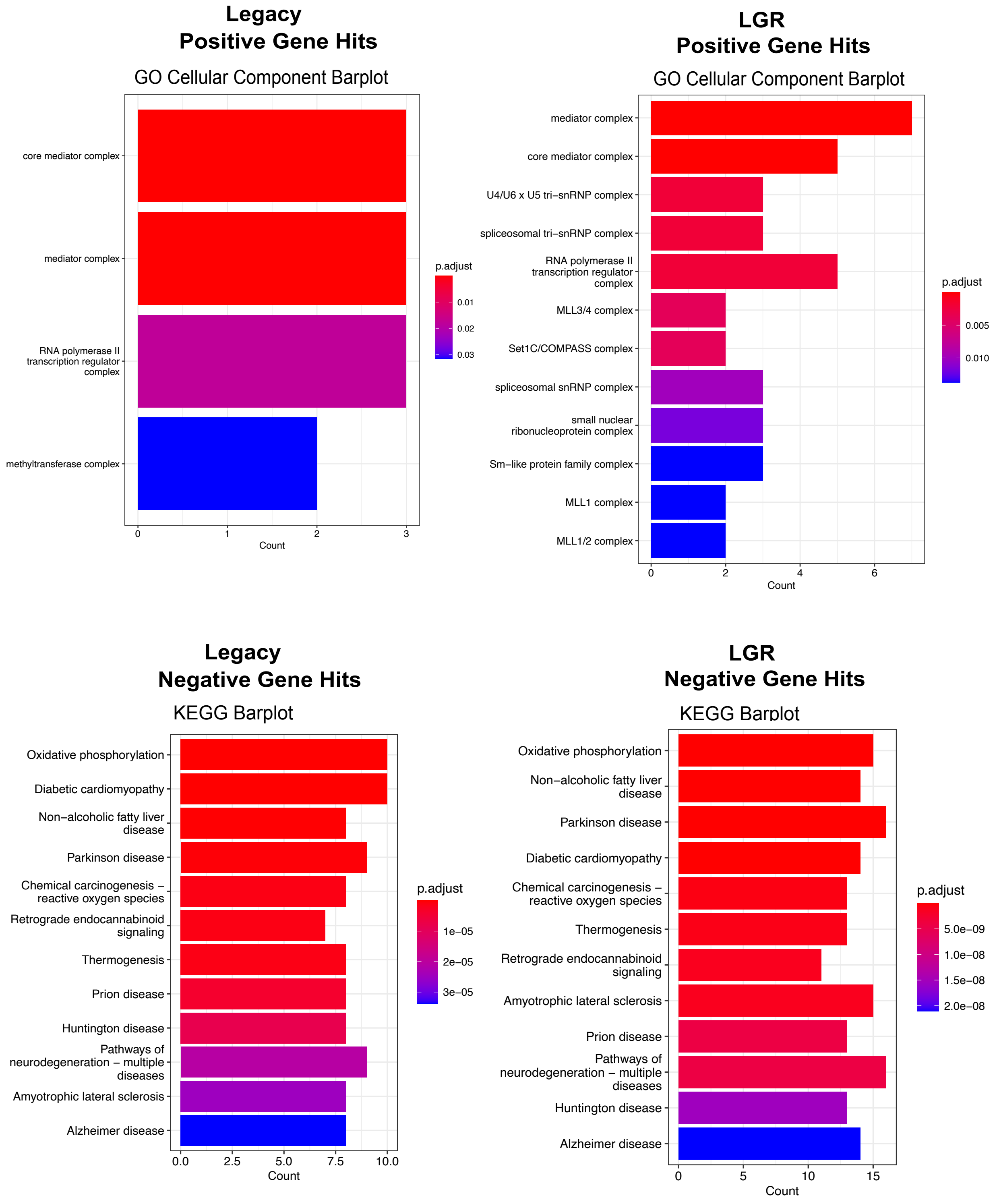

Fig S9. Bar plots ranking the top 12 positive and negative gene hits for the 100-fold cell coverage dasatinib survival screen performed with the legacy (left) and LGR (right) libraries. Gene hits are ranked by p-value assigned by ClusterProfiler (53,56).

# Fig. S10

A

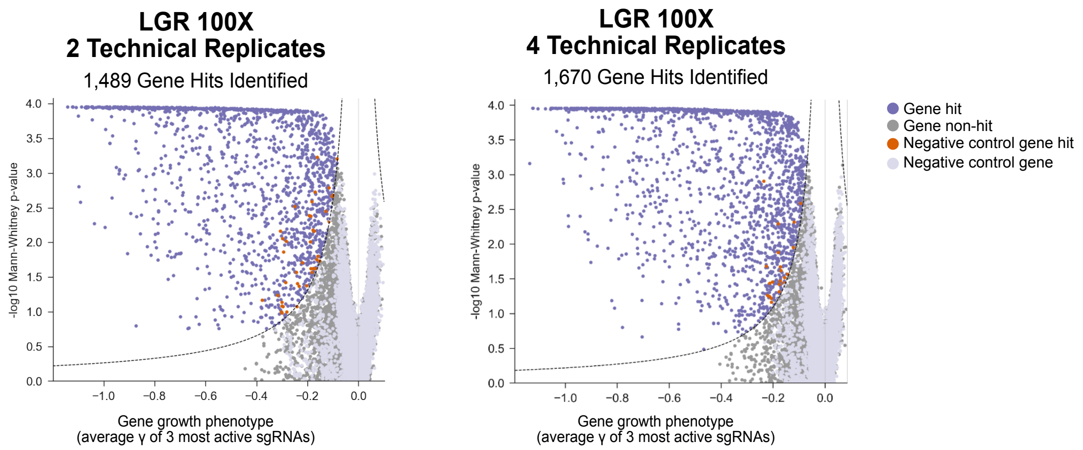


B

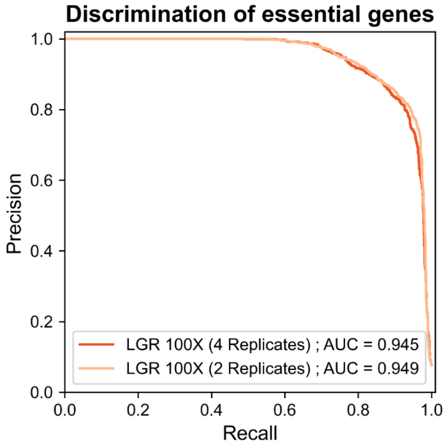


Fig S10. (A) The total number of essential genes identified in the LGR 100-fold cell screen increases as more technical replicates are processed. (B) Precision-recall analysis of the essential genes identified in the LGR 100x screen using either 2 or 4 technical replicates using BAGEL2 (23,24) to measure screening quality. The area under the curve (AUC) for the screen with 2 technical replicates was 0.949 and for 4 technical replicates was 0.945.

## Table S8

| Screening Coverage | Number of Infected Cells for 100,000 Guide Library | Number of Cells to Transduce with a 25 % Infection | Suspension Cell Line Culture Volume for 1 Sample *(with 3 technical replicates at ~500,000 cells/mL)* | Suspension Cell Line Shaker Flask Size for 1 Sample | Adherent Cell Line Flask Size for 1 Sample |
|---|---|---|---|---|---|
| 10X | 1 million | 4 million | 6 mL *(30 mL minimum 125mL culture volume)* | 125 mL | T25 |
| 50X | 5 million | 20 million | 30 mL | 125 mL | T75 |
| 100X | 10 million | 40 million | 60 mL | 125 mL | T175 |
| 200X | 20 million | 80 million | 120 mL | 250 mL | T175 |
| 500X | 50 million | 200 million | 300 mL | 500 mL | Two T225 |
| 1000X | 100 million | 400 million | 600 mL | 1 L | Four T225 |

Table S8. A table demonstrating the quantity of materials necessary to perform genome-wide screens at increasing coverages (10 to 1000-fold cell coverage). Labware recommendations were acquired from ThermoFisher.
