# Supplementary material for: Compact CRISPR genetic screens enabled by improved guide RNA library cloning
Source: Genome Biol. 2024 Jan 19;25:25. doi: 10.1186/s13059-023-03132-3 (PMC10797759; doi:10.1186/s13059-023-03132-3)
Supplement: Supplementary file 15 — Additional file 15. [file 13059_2023_3132_MOESM15_ESM.docx]

Review History

**First round of review**

**Reviewer 1**

**Were you able to assess all statistics in the manuscript, including the appropriateness of statistical tests used?**

No.

**Were you able to directly test the methods?**

No.

**Comments to author:**

In this manuscript, the authors report an improved method for cloning pooled sgRNA libraries that results in markedly more even representation of elements. The authors showcase the utility of this method by re-generating existing sgRNA libraries and then conducting pooled screens at much lower coverage while retaining sensitivity to identify genes that impact a phenotype of interest. This work is an important step toward making CRISPR-based genetic screens more accessible. In addition, this work will likely inform generation of other types of pooled libraries as well and thus will have broader impacts. I found the manuscript and results easy to follow. The authors also include a detailed protocol that will make it easy for readers to implement this protocol on their own. I find this work to be worthy of publication, although I have a few suggestions for improving the manuscript and the impact of the work.

My major comment is that the dasitinib screens, although well executed, thus far seem like a missed opportunity to dig deeper into the advantages of the library cloning method and the use of more even libraries. In particular the major benchmark right now is the number of hits at different FDRs, but there can be all sorts of technical reasons for different numbers of hits beyond lower skew in the library. Might there be ways to link the lower skew more explicitly to improved screen sensitivity? For example, the authors could compare how many sgRNAs are filtered out during data processing with standard thresholds for the two libraries and then ask which of the ones that are recovered only in the lower skew libraries contribute to the gene-level phenotypes. Or perhaps the authors could compare phenotype strength and relative library abundance for all sgRNAs. There are many other possibilities - these are just some ideas.

Second, although the newly cloned libraries clearly have much lower skew than the original CRISPRia v2 libraries and other CRISPR libraries, I have some suggestions for making the comparisons more clear:

- the data used for the comparison in figure 2C seem to be somewhat undersequenced. In particular it is difficult to tell if the elements with zero counts are true zeros. Were those elements included in skew calculations? If possible, it might be worthwhile obtaining ~10-fold deeper sequencing to estimate the skew ratios more accurately.

- for the data presented in figure 2D, I might recommend making line graphs plotting the skew vs percentile for the different libraries

- my understanding is that the 7 sublibraries of the CRISPRia v2 libraries were cloned independently and thus have different skews. I think it would be appropriate to split the comparison of skew into the individual sublibraries.

- I feel that the authors could absolutely emphasize more explicitly that they cloned all 100,000 elements in a single reaction, which makes the low skew in the libraries all the more impressive.

Minor comments:

1. The authors emphasize ordering oligos in both forward and reverse complement orientations as a major innovation, in the introduction and elsewhere. My understanding is that this is already routinely done in many other instances, although perhaps often not acknowledged in methods sections. I think there is enormous value in the explicit comparison the authors have done here, but I would recommend not overstating the significance of the methodological innovation. Instead the authors could note that they report an explicit comparison of this parameter.

2. Regarding the polymerase comparison: many older protocols have used polymerases other than the ones used in this manuscript - although Q5 clearly performs well, it may be worth adding a note to this respect in the manuscript

3. Lines 168 onward (comparison of AUCs in essential gene identification): the improvement in AUC is modest and could be the result of lots of factors, especially because the screens were not performed side by side and in different cell isolates. I would recommend toning down the claim that the new protocol results in improved identification of essential genes. Instead the authors could emphasize that this cloning protocol clearly yields libraries that identify essential genes with high precision (and ideally accuracy).

4. Lines 185-196 and Figure 4B: could the authors provide an explanation for why element representation is more correlated for the legacy library? Are the highly abundant elements in the legacy library skewing these correlations, and if so might a different estimate of correlation be more appropriate (e.g. spearman)? In addition, please provide the metric of correlation used.

5. Dasatinib screen: the LGR library seems to shine particularly in identifying genes whose knockdown benefits survival in the presence of dasatinib. Could the authors provide an explanation for this? For example, are these additional hits generally genes that are essential and thus whose sgRNAs usually have low abundance by the end of a screen?

6. Figure S1: the right side of the BstXI site is misannotated by 1 base

7. Figure S9A: it might be more informative to plot the cumulative cell doublings rather than the cell viability.

8. Pertaining to the major comment above, please make sure all correlation metrics are clearly described.

**Reviewer 2**

**Were you able to assess all statistics in the manuscript, including the appropriateness of statistical tests used?**

Yes: The statistics were standard for CRISPR functional genomics studies.

**Were you able to directly test the methods?**

No.

**Comments to author:**

Heo & Enriquez et al. report an optimized protocol for cloning genome-wide CRISPR sgRNA libraries for CRISPRi/a-based genetic perturbations. Previous libraries suffered from lack of uniformity per unique sgRNA elements and required high cell numbers to achieve high quality phenotypic quantifications. The manuscript describes multiple optimization steps to improve sgRNA uniformity, including technical experimental tricks such as reduced temperatures for sgRNA oligo annealing, gel electrophoresis, and elution from columns. Together, the authors' molecular optimizations led to highly uniform libraries (highlighted beautifully in Figure 2). Perhaps the biggest impact of the library optimizations is the ability to reduce the number of cells down to 50-100x per unique sgRNA element - a major advancement in CRISPR-based functional genomics screens. I envision that the libraries will be used widely and will be an important resource for the biomedical research community.

The manuscript is well-written, and I support its publication with no further experiments. Below are points that may be addressed in the manuscript of response to reviews:

1. All of the experiments were performed in K562 cells and the new libraries were benchmarked only with CRISPRi datasets. Though I can understand that the results will be predictable, testing the optimized CRISPRa libraries would strengthen the manuscript especially as CRISPRa screens can yield more variability than CRISPRi.

2. In Figures 3 and S7, the authors compare the unique hits between the Horlbeck (Legacy) and LGR screen phenotypes. The Legacy library yielded more gene hits than the LGR. Can the authors speculate on whether the difference in the number of gene hits is typical for CRISPR screens performed at independent times or whether the difference is indeed due to the LGR library design? It is difficult to gauge whether the authors' claims about the differing gene hits across screens is inherent noise in CRISPR screen data.

3. Along this topic, it could be helpful to plot the phenotype score of unique hits from the Legacy screen (476 according to Fig. 3D) against the phenotype score from the LGR screens of the same gene set. I am mostly curious whether the Legacy unique hits gave no growth defect with the LGR library (and vice versa for the unique LGR hits).

4. Recent data have emerged highlighting the adverse effects of double stranded DNA breaks from Cas9 genome editing (see below). It may add impact to the manuscript to highlight the advantage of CRISPRi screens over Cas9 nucleases. This could be expanded to discussion/citations of papers showing cell toxicity of genome editing in stem cells.

1. Tsuchida, C. et al. Migitation of chromosome loss in clinical CRISPR-Cas9-engineered T cells. bioRxiv (2023). https://www.biorxiv.org/content/10.1101/2023.03.22.533709v1

2. Lazar, N. et al. High-resolution genome-wide mapping of chromosome-arm-scale truncations induced by CRISPR-Cas9 editing. bioRxiv (2023).

https://www.biorxiv.org/content/10.1101/2023.04.15.537038v1

5. Another citation worth discussing is the potential advantage over the recent compact Weissman two protospacer sgRNA system, which are prone to high recombination rates between protospacers. Since the Weissman library is a one element per gene design, it is not possible to compute a p-value compared to the multiple elements per gene design of the LGR/Legacy libraries. An added advantage of the LGR libraries would be to minimize cell numbers while being able to compute statistics.

https://elifesciences.org/articles/81856

**Authors Response**

**Point-by-point responses to the reviewers’ comments:**

**Reviewer #1:** In this manuscript, the authors report an improved method for cloning pooled sgRNA libraries that results in markedly more even representation of elements. The authors showcase the utility of this method by re-generating existing sgRNA libraries and then conducting pooled screens at much lower coverage while retaining sensitivity to identify genes that impact a phenotype of interest. This work is an important step toward making CRISPR-based genetic screens more accessible. In addition, this work will likely inform generation of other types of pooled libraries as well and thus will have broader impacts. I found the manuscript and results easy to follow. The authors also include a detailed protocol that will make it easy for readers to implement this protocol on their own. I find this work to be worthy of publication, although I have a few suggestions for improving the manuscript and the impact of the work.

1. My major comment is that the dasitinib screens, although well executed, thus far seem like a missed opportunity to dig deeper into the advantages of the library cloning method and the use of more even libraries. In particular the major benchmark right now is the number of hits at different FDRs, but there can be all sorts of technical reasons for different numbers of hits beyond lower skew in the library. Might there be ways to link the lower skew more explicitly to improved screen sensitivity? For example, the authors could compare how many sgRNAs are filtered out during data processing with standard thresholds for the two libraries and then ask which of the ones that are recovered only in the lower skew libraries contribute to the gene-level phenotypes. Or perhaps the authors could compare phenotype strength and relative library abundance for all sgRNAs. There are many other possibilities - these are just some ideas.

*Response: We thank the reviewer for this feedback. We examined why there are more unique gene hits in the LGR library and focused on the three Mediator complex genes in the dasatinib screen that only came up as hits using the LGR library. We have created a new panel 6B showing the abundance of each guide for the three unique hits, which shows knockdown of these genes results in a growth defect in the absence of dasatinib. This likely causes a bottlenecking event before dasatinib treatment. With increased uniformity of the LGR library, we get more consistent values for each guide compared to the legacy library despite this bottleneck. We added the following line to the results section: “Examining the individual guide abundance in the unique hits, (Figure 6B), we observe that silencing of the Mediator components results in a growth defect and bottleneck for these guides. However, knockdown of these components promotes resistance to dasatinib treatment. The uniform abundance of individual guides in the LGR screen results in less variability after the treatment bottleneck.” This additional analysis therefore provides a direct link between lower skew and improved ability to call gene hits from pooled screens.*

2. Second, although the newly cloned libraries clearly have much lower skew than the original CRISPRia v2 libraries and other CRISPR libraries, I have some suggestions for making the comparisons more clear: the data used for the comparison in figure 2C seem to be somewhat undersequenced. In particular it is difficult to tell if the elements with zero counts are true zeros. Were those elements included in skew calculations? If possible, it might be worthwhile obtaining ~10-fold deeper sequencing to estimate the skew ratios more accurately.

*Response: We thank the reviewer for this comment. To clarify this point, we have added an additional table [Additional File 3 – Figure 2 Sequencing Stats.xlsx] with sequencing statistics. Libraries were sequenced to a depth of ~500-2000 fold coverage which gives good representation. For both the CRISPRi and CRISPRa plasmid libraries, the legacy library actually received ~1.4 or 3.8 times more reads than the LGR library. They appear under sequenced because of the large skew and number of dropouts.*

3. for the data presented in figure 2D, I might recommend making line graphs plotting the skew vs percentile for the different libraries

*Response: We thank the reviewer for this suggestion and agree that this is a more effective way to communicate the results. We also implemented this suggestion for figure [2C] and the transduction titration experiment [4C]. We split the drop out data into a similar figure [4D].*

4. my understanding is that the 7 sublibraries of the CRISPRia v2 libraries were cloned independently and thus have different skews. I think it would be appropriate to split the comparison of skew into the individual sublibraries.

*Response: This is correct. We created a table where we split the guides insilico into the seven sublibraries but we don’t feel the plots add to the discussion. The table has been included at the end of the review but we have added the line “Even more impressive, the legacy library was cloned as seven smaller subpools, compared to a single large pool for our library.”*

5. I feel that the authors could absolutely emphasize more explicitly that they cloned all 100,000 elements in a single reaction, which makes the low skew in the libraries all the more impressive.

*Response: We thank the reviewer for this comment and have added the following text to the relevant section “Even more impressive, the legacy library was cloned as seven smaller subpools compared to a single large pool for our library.”*

Minor comments:
6. The authors emphasize ordering oligos in both forward and reverse complement orientations as a major innovation, in the introduction and elsewhere. My understanding is that this is already routinely done in many other instances, although perhaps often not acknowledged in methods sections. I think there is enormous value in the explicit comparison the authors have done here, but I would recommend not overstating the significance of the methodological innovation. Instead the authors could note that they report an explicit comparison of this parameter.

*Response: We agree with this comment. We have added the comment that other groups already order oligos in both orientations, but we were unaware of any actual data demonstrating the practical utility of this strategy.*
7. Regarding the polymerase comparison: many older protocols have used polymerases other than the ones used in this manuscript - although Q5 clearly performs well, it may be worth adding a note to this respect in the manuscript

*Response: We agree with the comment and have added the following line "While we used NEB Q5 in this study, there are other NGS-optimized polymerases that could provide even better performance.”*

8. Lines 168 onward (comparison of AUCs in essential gene identification): the improvement in AUC is modest and could be the result of lots of factors, especially because the screens were not performed side by side and in different cell isolates. I would recommend toning down the claim that the new protocol results in improved identification of essential genes. Instead the authors could emphasize that this cloning protocol clearly yields libraries that identify essential genes with high precision (and ideally accuracy).

*Response: We thank the reviewer for this comment and agree. We have deleted the line “This demonstrates that the improvements we made in the cloning protocol resulted in an improvement in the discrimination of essential genes, thereby creating a high-quality screening library.”*

*We feel the improvement in AUC is warranted in the Dasatinib screen since it was performed with the same cell line under the same conditions (drug selection, screen duration, etc) in parallel at the same time but welcome additional feedback from the reviewer.*

9. Lines 185-196 and Figure 4B: could the authors provide an explanation for why element representation is more correlated for the legacy library? Are the highly abundant elements in the legacy library skewing these correlations, and if so might a different estimate of correlation be more appropriate (e.g. spearman)? In addition, please provide the metric of correlation used.

*Response: We believe the reviewers hypothesis is correct, the higher skews in the legacy library are skewing these correlations. Since the legacy library has a less uniform and more widely distributed guide abundance, it should result in a stronger correlation than the LGR library as long as we are not under sampling. A useful thought exercise is to imagine a library with a perfectly even distribution where every guide has the name number of counts. Replicates will have a very poor correlation. We believe this panel does not add to the discussion and have removed it. Additionally, we tried visualizing with a Spearman rank correlation and see similar results to the Pearson linear correlation. The Spearman correlation figure has been added to the end of this response for the reviewer but has not been added to the manuscript.*

*We have also gone through the text and made sure to specify which correlation metric has been used. We used linear regression for most of our analyses except for the correlation that came out of MAGeCK, which uses Pearson correlations.*

10. Dasatinib screen: the LGR library seems to shine particularly in identifying genes whose knockdown benefits survival in the presence of dasatinib. Could the authors provide an explanation for this? For example, are these additional hits generally genes that are essential and thus whose sgRNAs usually have low abundance by the end of a screen?

*Response: We thank the review for this comment. This is similar feedback to the first comment from reviewer 1, which we have addressed by creating a new panel 6B. This panel includes the abundance of each guide for the three unique hits in the dasatinib screen, which shows knockdown of these genes results in a growth defect in the absence of dasatinib, as described above in response to comment 1.*

11. Figure S1: the right side of the BstXI site is misannotated by 1 base

*Response: We thank the review for catching this. We have corrected the figure.*

12. Figure S9A: it might be more informative to plot the cumulative cell doublings rather than the cell viability.

*Response: We have plotted this and included it at the end of this reponse but feel that since this is a drug selection screen, plotting doubling for drug treatment will be tricky because of the growth inhibition. We don’t think it adds to the manuscript but have included a chart of doubling for each of the samples. While the numbers are slightly different for the samples, they are within one cell doubling for all paired samples.*

13. Pertaining to the major comment above, please make sure all correlation metrics are clearly described.

*Response: We thank the reviewer for noting this and have described which correlation metric was used in each analysis*.

**Reviewer #2:** Heo & Enriquez et al. report an optimized protocol for cloning genome-wide CRISPR sgRNA libraries for CRISPRi/a-based genetic perturbations. Previous libraries suffered from lack of uniformity per unique sgRNA elements and required high cell numbers to achieve high quality phenotypic quantifications. The manuscript describes multiple optimization steps to improve sgRNA uniformity, including technical experimental tricks such as reduced temperatures for sgRNA oligo annealing, gel electrophoresis, and elution from columns. Together, the authors' molecular optimizations led to highly uniform libraries (highlighted beautifully in Figure 2). Perhaps the biggest impact of the library optimizations is the ability to reduce the number of cells down to 50-100x per unique sgRNA element - a major advancement in CRISPR-based functional genomics screens. I envision that the libraries will be used widely and will be an important resource for the biomedical research community.

The manuscript is well-written, and I support its publication with no further experiments. Below are points that may be addressed in the manuscript of response to reviews:

1. All of the experiments were performed in K562 cells and the new libraries were benchmarked only with CRISPRi datasets. Though I can understand that the results will be predictable, testing the optimized CRISPRa libraries would strengthen the manuscript especially as CRISPRa screens can yield more variability than CRISPRi.

*Response: We thank the reviewer for this comment and agree with it. While we feel that performing CRISPRa screens is outside the scope of this methods paper, we have used the same methods described to produce a genome-wide CRISPRa library with similarly low skew and are currently utilizing it library in other projects that we will publish in the future. This CRISPRa library will also be made available on request.*

2. In Figures 3 and S7, the authors compare the unique hits between the Horlbeck (Legacy) and LGR screen phenotypes. The Legacy library yielded more gene hits than the LGR. Can the authors speculate on whether the difference in the number of gene hits is typical for CRISPR screens performed at independent times or whether the difference is indeed due to the LGR library design? It is difficult to gauge whether the authors' claims about the differing gene hits across screens is inherent noise in CRISPR screen data.

*Response: We agree with the reviewer. The differing number of hits is reasonable given the screens were performed years apart by different individuals and different sources for the parental K562 cell line. This is one reason we performed additional experiments with both the Legacy and LGR libraries in parallel later in the paper. We have expanded the sentence in the discussion to further highlight the fact that the Legacy screen was performed years earlier in a different lab and engineered cell line. “This is expected since the same library and cell doublings were used compared to the legacy screen. Additionally, the legacy screen was performed years earlier in a different lab and parental K562 line.”*

3. Along this topic, it could be helpful to plot the phenotype score of unique hits from the Legacy screen (476 according to Fig. 3D) against the phenotype score from the LGR screens of the same gene set. I am mostly curious whether the Legacy unique hits gave no growth defect with the LGR library (and vice versa for the unique LGR hits).

*Response: We have plotted the phenotype scores of the unique legacy hits vs the scores in the LGR screen and have added this to the supplemental figures. We also plotted the phenotype scores of the unique gene hits in the LGR screen vs their value in the legacy screen. There is a weak correlation. These phenotype correlation plots have been added to supplemental figure S7, where we highlight that most of the unique hits in each screen fall just outside the cutoff and are likely noisy results. A few additional lines have been added to the results section of the manuscript.*

4. Recent data have emerged highlighting the adverse effects of double stranded DNA breaks from Cas9 genome editing (see below). It may add impact to the manuscript to highlight the advantage of CRISPRi screens over Cas9 nucleases. This could be expanded to discussion/citations of papers showing cell toxicity of genome editing in stem cells.

1. Tsuchida, C. et al. Migitation of chromosome loss in clinical CRISPR-Cas9-engineered T cells. bioRxiv (2023). Lazar, N. et al. High-resolution genome-wide mapping of chromosome-arm-scale truncations induced by CRISPR-Cas9 editing. bioRxiv (2023).

*Response: We thank the review for this helpful suggestion and have added a line incorporating these two citations in the results section. We feel further discussion talking about toxicity in stem cells, while important, is outside the scope of this manuscript.*

5. Another citation worth discussing is the potential advantage over the recent compact Weissman two protospacer sgRNA system, which are prone to high recombination rates between protospacers. Since the Weissman library is a one element per gene design, it is not possible to compute a p-value compared to the multiple elements per gene design of the LGR/Legacy libraries. An added advantage of the LGR libraries would be to minimize cell numbers while being able to compute statistics.
<https://elifesciences.org/articles/81856>

*Response: We thank the review for this very helpful suggestion that adds to the manuscript. We have added the following text to the end of the discussion “New compact dual-guide libraries [https://doi.org/10.7554/eLife.81856] have reduced the number of elements to as few as one per gene. This allows lower usage of cells in single-gene screens as well as the ability to perform combinatorial screens. However, this precludes the calculation of p-values to filter hits. In single gene screens with our library at 50x cell coverage minimizes the number of cells to similar levels as the dual-guide library,* *especially when factoring in the number of cells transduced with recombined lentiviral particles rates (~30%) of the dual guide systems.”*

**Figures for Reviewers**

1. Sublibrary skews

| **CRISPRi - LGR** | **# Guides** | **90/10** | **95/5** | **98/2** | **99/1** | **99.5/0.5** |
| --- | --- | --- | --- | --- | --- | --- |
| **Entire Library** | 103,074 | 1.99 | 2.36 | 2.86 | 3.25 | 3.73 |
| **h1** | 12,930 | 2.00 | 2.37 | 2.86 | 3.28 | 3.67 |
| **h2** | 16,197 | 1.99 | 2.36 | 2.84 | 3.25 | 3.73 |
| **h3** | 16,699 | 1.99 | 2.39 | 2.89 | 3.26 | 3.71 |
| **h4** | 12,161 | 1.99 | 2.36 | 2.88 | 3.27 | 3.79 |
| **h5** | 12,509 | 1.99 | 2.36 | 2.87 | 3.28 | 3.75 |
| **h6** | 13,090 | 1.97 | 2.32 | 2.80 | 3.15 | 3.66 |
| **h7** | 19,488 | 1.97 | 2.33 | 2.81 | 3.22 | 3.72 |
|  |  |  |  |  |  |  |
| **CRISPRi - Legacy** | **# Guides** | **90/10** | **95/5** | **98/2** | **99/1** | **99.5/0.5** |
| **Entire Library** | 103,074 | 5.11 | 10.27 | 33.29 | 171.38 | inf |
| **h1** | 12,930 | 4.97 | 8.61 | 19.00 | 33.77 | 102.39 |
| **h2** | 16,197 | 3.27 | 5.03 | 9.87 | 17.60 | 36.77 |
| **h3** | 16,699 | 2.91 | 4.24 | 6.90 | 11.04 | 19.07 |
| **h4** | 12,161 | 3.24 | 4.91 | 8.46 | 13.88 | 27.36 |
| **h5** | 12,509 | 4.51 | 6.87 | 11.98 | 19.05 | 33.90 |
| **h6** | 13,090 | 8.78 | 21.25 | 130.06 | inf | inf |
| **h7** | 19,488 | 12.89 | 44.59 | inf | inf | inf |
|  |  |  |  |  |  |  |
| **CRISPRa-LGR** | **# Guides** | **90/10** | **95/5** | **98/2** | **99/1** | **99.5/0.5** |
| **Entire Library** | 101,250 | 1.98 | 2.46 | 3.17 | 3.91 | 5.10 |
| **h1** | 12,751 | 2.01 | 2.51 | 3.22 | 3.94 | 5.47 |
| **h2** | 15,918 | 2.01 | 2.51 | 3.20 | 3.99 | 5.26 |
| **h3** | 16,277 | 1.97 | 2.42 | 3.14 | 3.85 | 4.95 |
| **h4** | 11,909 | 1.99 | 2.49 | 3.27 | 4.15 | 5.61 |
| **h5** | 12,207 | 1.98 | 2.45 | 3.26 | 4.08 | 5.78 |
| **h6** | 12,906 | 1.96 | 2.41 | 3.07 | 3.66 | 4.58 |
| **h7** | 19,282 | 1.97 | 2.41 | 3.07 | 3.80 | 4.54 |
|  |  |  |  |  |  |  |
| **CRISPRa-Legacy** | **# Guides** | **90/10** | **95/5** | **98/2** | **99/1** | **99.5/0.5** |
| **Entire Library** | 101,250 | 2.80 | 3.97 | 6.57 | 10.99 | 23.12 |
| **h1** | 12,751 | 3.04 | 4.33 | 7.11 | 11.42 | 19.97 |
| **h2** | 15,918 | 2.76 | 4.00 | 6.78 | 11.66 | 29.54 |
| **h3** | 16,277 | 2.74 | 4.12 | 7.65 | 14.83 | 32.68 |
| **h4** | 11,909 | 2.55 | 3.57 | 6.26 | 10.42 | 16.50 |
| **h5** | 12,207 | 2.90 | 4.13 | 6.63 | 10.61 | 53.46 |
| **h6** | 12,906 | 2.08 | 2.67 | 3.55 | 4.52 | 7.25 |
| **h7** | 19,282 | 2.77 | 3.95 | 6.86 | 11.65 | 22.00 |

2. Spearman correlation of Titration T0 samples


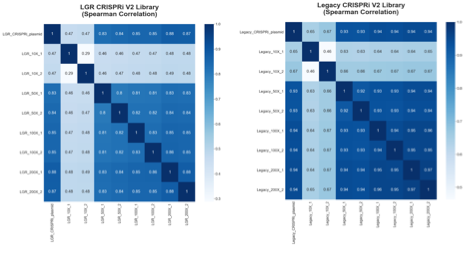


3. Dasatinib screen cell doubling plot

**Second round of review**

**Reviewer 1**

The authors have generally addressed my comments. I congratulate them on an excellent body of work.

My only remaining comment is that the description of the skew in the legacy library feels like a misrepresentation and creates unwarranted concern for current and previous users of the legacy library. I would strongly recommend reporting the skew values in Figure 2C broken down by the sublibraries. Reporting the skew in this fashion also more accurately represents the utility of the new cloning approach. Indeed, this would clearly documents that the new cloning approach both outperforms previous approaches even in the best-case scenario and also avoids previous issues with heterogeneity across sublibraries.

Two minor points:

- the labels in figure 6B are not aligned

- Line 193: Should be Figure S7B, not S2B

**Authors Response**

**Point-by-point responses to the reviewers’ comments:**

Reviewer #1:

The authors have generally addressed my comments. I congratulate them on an excellent body of work.

My only remaining comment is that the description of the skew in the legacy library feels like a misrepresentation and creates unwarranted concern for current and previous users of the legacy library. I would strongly recommend reporting the skew values in Figure 2C broken down by the sublibraries. Reporting the skew in this fashion also more accurately represents the utility of the new cloning approach. Indeed, this would clearly documents that the new cloning approach both outperforms previous approaches even in the best-case scenario and also avoids previous issues with heterogeneity across sublibraries.

Two minor points:

- the labels in figure 6B are not aligned

- Line 193: Should be Figure S7B, not S2B

*Response: The reviewer comments about the sublibrary skew ratios was added to the text the figures. Figure alignment was also corrected. We thank the reviewer for the useful feedback.*
